# Identifying Transcriptional Regulatory Modules Among Different Chromatin States in Mouse Neural Stem Cells

**DOI:** 10.3389/fgene.2018.00731

**Published:** 2019-01-15

**Authors:** Sharmi Banerjee, Hongxiao Zhu, Man Tang, Wu-chun Feng, Xiaowei Wu, Hehuang Xie

**Affiliations:** ^1^Bradley Department of Electrical and Computer Engineering, Virginia Tech, Blacksburg, VA, United States; ^2^Biocomplexity Institute of Virginia Tech, Blacksburg, VA, United States; ^3^Department of Statistics, Virginia Tech, Blacksburg, VA, United States; ^4^Department of Computer Science, Virginia Tech, Blacksburg, VA, United States; ^5^Department of Biomedical Sciences and Pathobiology, Virginia-Maryland College of Veterinary Medicine, Blacksburg, VA, United States; ^6^Department of Biological Sciences, Virginia Tech, Blacksburg, VA, United States; ^7^School of Neuroscience, Virginia Tech, Blacksburg, VA, United States

**Keywords:** transcription factor, regulatory network, Poisson process, chromatin states, neural stem cell

## Abstract

Gene expression regulation is a complex process involving the interplay between transcription factors and chromatin states. Significant progress has been made toward understanding the impact of chromatin states on gene expression. Nevertheless, the mechanism of transcription factors binding combinatorially in different chromatin states to enable selective regulation of gene expression remains an interesting research area. We introduce a nonparametric Bayesian clustering method for inhomogeneous Poisson processes to detect heterogeneous binding patterns of multiple proteins including transcription factors to form regulatory modules in different chromatin states. We applied this approach on ChIP-seq data for mouse neural stem cells containing 21 proteins and observed different groups or modules of proteins clustered within different chromatin states. These chromatin-state-specific regulatory modules were found to have significant influence on gene expression. We also observed different motif preferences for certain TFs between different chromatin states. Our results reveal a degree of interdependency between chromatin states and combinatorial binding of proteins in the complex transcriptional regulatory process. The software package is available on Github at - https://github.com/BSharmi/DPM-LGCP.

## 1. Introduction

Transcription factors (TFs) and other proteins that bind to specific DNA sequences play key roles in the regulation of gene expression. Binding locations of a protein of interest can be determined with chromatin immunoprecipitation followed by sequencing (ChIP-seq). This produces millions of short reads covering the protein-DNA binding sites across the genome. Several computational tools have been developed to identify these binding locations from ChIP-seq data. Widely used among these is MACS2 (Feng et al., 2012) which can identify transcription factor binding regions or “peaks.” Recently, efforts have been devoted to integrate multiple ChIP-seq datasets to uncover protein-protein interactions. SignalSpider (Wong et al., 2015) uses Gaussian mixture model to reveal regions co-regulated by multiple TFs. Sharmin et al. identified cell-type specific TF binding events (Sharmin et al., 2016) using ensemble model. Cha and Zhou developed a method based on inhomogeneous Poisson processes and Ripley's K-function that detects pairwise TF clustering and ordering patterns (Cha and Zhou, 2014).

Recent studies have also revealed new insights into the interplay between proteins, specifically TFs and histone marks that define chromatin states. Most TFs bind to open chromatin regions that are highly accessible and nucleosome-depleted. Such chromatin regions are often enriched with specific histone modifications in promoters and enhancers, such as H3K4me1 and H3K27ac marks. It has been found that histone-modification-dependent TF binding is protein family specific (Sugathan and Waxman, 2013; Liu et al., 2015, 2016; Xin and Rohs, 2018). In addition, a small number of TFs act as pioneers with the ability to reach inaccessible chromatin regions and shape the chromatin landscape to facilitate the binding of other TFs. ChIP-seq data from histone modifications have been used to partition the genome into different chromatin states using semi-automated genome annotation (SAGA) tools (Libbrecht et al., 2015). Early examples of the SAGA tools are HMMSeg (Day et al., 2007) and ChromHMM (Ernst and Kellis, 2012). Since then more sophisticated chromatin segmentation tools, Segway (Hoffman et al., 2012) and diHMM (Marco et al., 2017), were developed providing refined genome-wide map of the chromatin states. ChromHMM and diHMM use hidden Markov models while Segway applies a dynamic Bayesian network to segment the genome and identify distinct chromatin states. Segway and ChromHMM perform genome segmentation and classification at a single length scale while diHMM segments the genome at multiple length scales (narrow or broad corresponding to nucleosome-level states and domain-level states, respectively). We studied protein bindings through ChIP-seq data among different chromatin states in mice neural stem cells (detailed description of datasets provided in Supplementary Document Section 3.1). Our results showed several known co-binding rules such as NFIC-bHLH-SOX in Upstream Enhancer state and Poised Enhancer state (Mateo et al., 2015) and JMJD3-SMAD3 in all chromatin states (Estarás et al., 2012). We also showed that the regulatory effects of the predicted modules on proximal genes vary across chromatin states. Also, for certain classes of DNA binding proteins, the *de-novo* binding sequences compiled from ChIP-seq peaks were dependent on the chromatin states.

## 2. Materials and Methods

**Figure 1:**
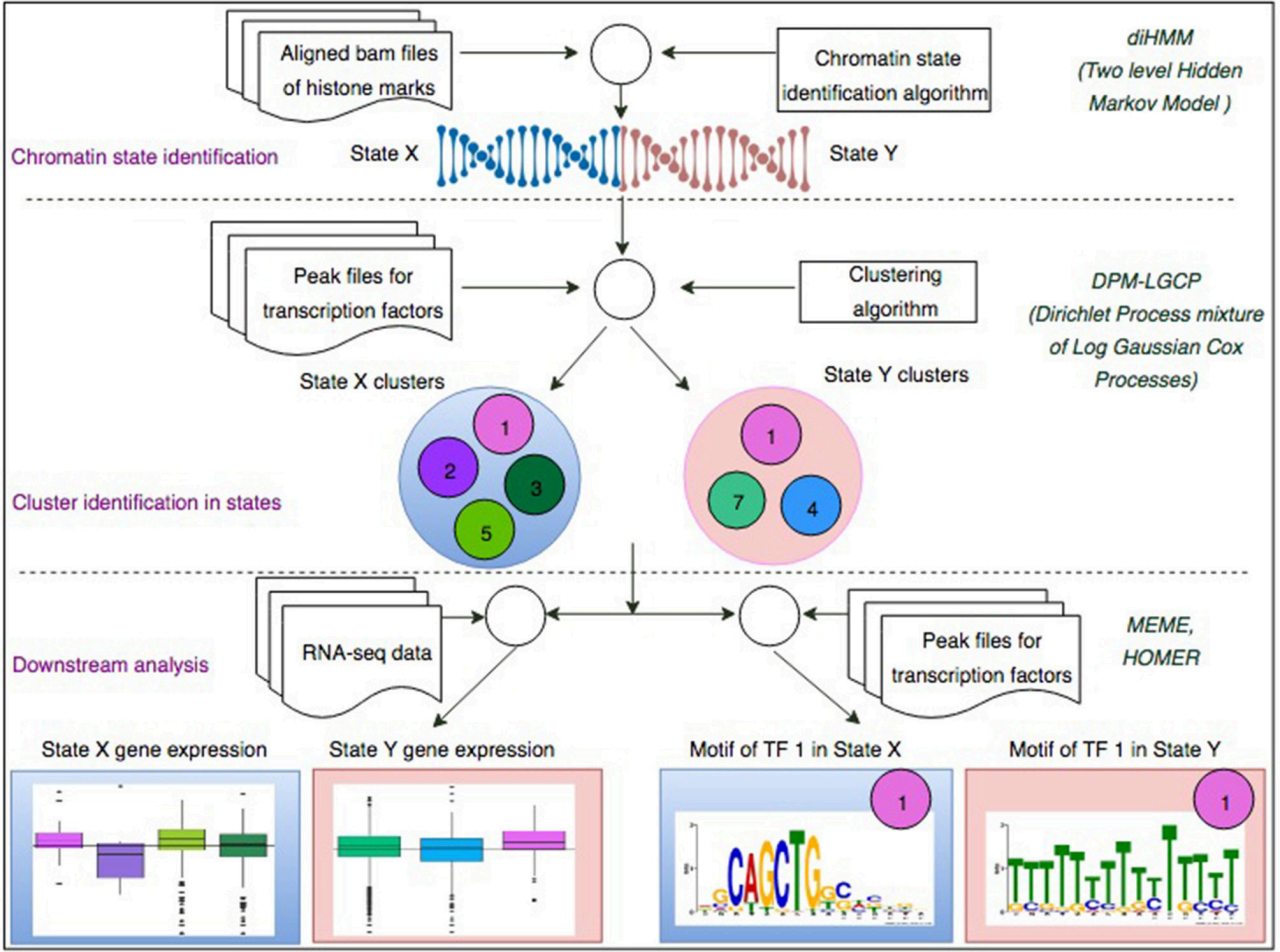
A two-step process to identify chromatin-state-specific transcriptional regulatory modules. In the first step, uniquely aligned bam files of histone marks are used along with the diHMM software to segment the genome and identify distinct chromatin states (illustrated by State X and State Y). In the second step, using the identified chromatin states from the previous step and ChIP-seq peak files for different TFs, the proposed Bayesian clustering method is applied to identify transcriptional regulatory modules within each chromatin state. In downstream analyses, proximal (± 2 kb from TSS) genes are used to compare the TPM expression level when regulated by individual TFs to that when regulated combinatorially by the predicted regulatory modules in step 2. Finally, using *de-novo* motif enrichment analysis, the binding sequences of the TFs are compared across different chromatin stats to study the effect of histone marks and co-factors on TF binding sequences.

In this paper we propose a two-step process (Figure 1) to investigate how chromatin configurations may affect the binding affinity of proteins. In the first step, uniquely aligned BAM files containing genomic regions of histone marks and TFs are used along with the diHMM software to segment the genome and identify distinct chromatin states (illustrated by chromatin state examples X and Y). In the second step, using the identified chromatin states from the previous step and protein binding regions obtained from ChIP-seq (data used in this study were obtained from ChIP-Atlas; http://chip-atlas.org), a nonparametric Bayesian clustering method DPM-LGCP is applied to identify transcriptional regulatory modules within each chromatin state. In downstream analyses, proximal (± 2 kb from transcription start site genes are used to compare the Transcripts Per Kilobase Million or TPM expression level when regulated by individual proteins to that when regulated combinatorially by the predicted regulatory modules in step 2. Finally, using *de-novo* motif enrichment analysis, the binding sequences of the proteins are compared across different chromatin states to study the effect of histone marks and co-factors on motif preferences. Details of the datasets used in the study can be found in Supplementary Table S2.

### 2.1. Chromatin State Identification Through Genome Segmentation

diHMM (Marco et al., 2017) is a tool based on hidden Markov model that models the presence or absence of a histone mark to a high degree of accuracy. It segments and annotates the genome into different chromatin states at multiple length scales by modeling the genome wide distribution of histone marks. By default, diHMM has two scales of classification: (a) nucleosome level, with finer resolution chromatin state windows of around 200 base-pair (bp) length and (b) domain level, formed by stitching together similar nucleosome-level windows and having broader chromatin state windows extending over 100kbp-long regions. The domain-level states identified by diHMM are able to recapitulate known patterns in the chromatin literature and capture functional differences among diverse regulatory elements (Marco et al., 2017). The first step in identifying chromatin states is to binarize uniquely aligned BAM files. This is implemented in ChromHMM (Ernst and Kellis, 2012), a predecessor of diHMM. The diHMM software provides several nucleosome- and domain-level statistics including nucleosome-level emissions, combined nucleosome-level fold enrichments for each domain, fractional genome coverage of each nucleosome- and domain-level state, and nucleosome and domain state lengths. These statistics, together with the relative distance information of nucleosome- and domain-level states from transcription start site (TSS) and the enrichment of nucleosome-level states in genomic regions, were jointly analyzed to annotate each state to a biologically relevant functional category (details provided in RESULTS section).

### 2.2. Protein Binding Intensity Estimation Using Dirichlet Process Mixture of Log Gaussian Cox Processes (DPM-LGCP)

Binding regions of the proteins were obtained using MACS2 acting as inputs to our proposed clustering algorithm. Treating the center of each region as a binary binding event, we modeled binding events of each protein along the genome by an inhomogeneous Poisson process (*IP*). We chose this modeling strategy for the following reasons: (i) the event of each binding site falling into a minuscule interval is a rare event, independent of the events in other non-overlapping intervals, and (ii) the non-uniform distribution of the peaks at different genomic locations can be well characterized by the intensity function of the inhomogeneous Poisson process. For a protein with *n* binding site locations, we map these locations to points in a closed interval *D* on the real line, denoted by *S* = {*s*_1_, …, *s*_*n*_}. Following the inhomogeneous Poisson process model setting, the likelihood of observing *S* can be written as Simpson et al. (2016)

(1) $$\begin{array}{r} f\left(S|\lambda \left(s\right)\right)=\text{exp}\left\{|D|-\int_{D}\lambda \left(s\right)ds\right\}\overset{n}{\underset{j=1}{\prod }}\lambda \left(s_{j}\right), \end{array}$$

where |*D*| is the interval length and λ(*s*), *s* ∈ *D* is the intensity function. The Poisson process likelihood (1) provides the basis for nonparametric clustering of proteins based on their binding patterns, resulting in identification of modules of co-binding proteins that share similar regulatory functions. For a given ChIP-seq dataset of *N* proteins coming from *K* clusters (with *K* unknown), we assume that proteins in the same cluster share a common intensity function, distinct from those in other clusters. Under this assumption, we implement a Dirichlet process mixture of log Gaussian Cox process (DPM-LGCP) model that employs a Dirichlet process (DP) prior to the latent log intensity functions to facilitate clustering of the intensity functions. Let *S*_*i*_ denote the binding site locations of the *i*th protein, the DPM-LGCP model can be described as follows:

(2) $$\begin{array}{r} \begin{array}{c} S_{i}|\lambda_{i}\left(s\right)\sim IP\left(\lambda_{i}\left(s\right)\right),s\in D,\;i=1,\ldots ,N,\\ \log\left(\lambda_{i}\left(s\right)\right)=z_{i}\left(s\right),\;z_{i}\left(s\right)\sim G,\\ G\sim DP\left(m,G_{0}\right),\;G_{0}=GP\left(0,C_{\theta }\right), \end{array} \end{array}$$

where *G* is a random distribution with a DP prior. The DP prior is characterized by two parameters *m* and *G*_0_, where *m* is the precision parameter, and *G*_0_ is the base measure. The base measure *G*_0_ is assumed to be a Gaussian process with mean 0 and covariance kernel *C*_θ_(,), and θ contains parameters that control the shape of the covariance kernel. The introduction of this DP prior to the latent log intensity functions naturally facilitates clustering of the *N* point processes based on their intensity functions. With this model, neither the number of clusters nor *ad-hoc* distance measure between two point processes needs to be specified.

To overcome the difficulty of calculating the marginal likelihood of the point process *S*_*i*_, we employed an approximate but efficient posterior inference using the Integrated Nested Laplace Approximations (INLA) package (Rue et al., 2009; Simpson et al., 2016).

The INLA approximation of the LGCP transforms the continuous covariance kernel of *z*_*i*_(*s*) into a discrete precision matrix of the B-spline basis coefficients on a regular grid, which enables very fast covariance computation (Rue and Held, 2005). Finally, posterior inference on the assignment of proteins into clusters is performed through a Markov chain Monte Carlo (MCMC) algorithm using Neal's Gibbs sampler (Neal, 2000) (detailed description provided in the Supplementary Document).

## 3. Results

### 3.1. Genome Segmentation and Chromatin State Identification

**Figure 2:**
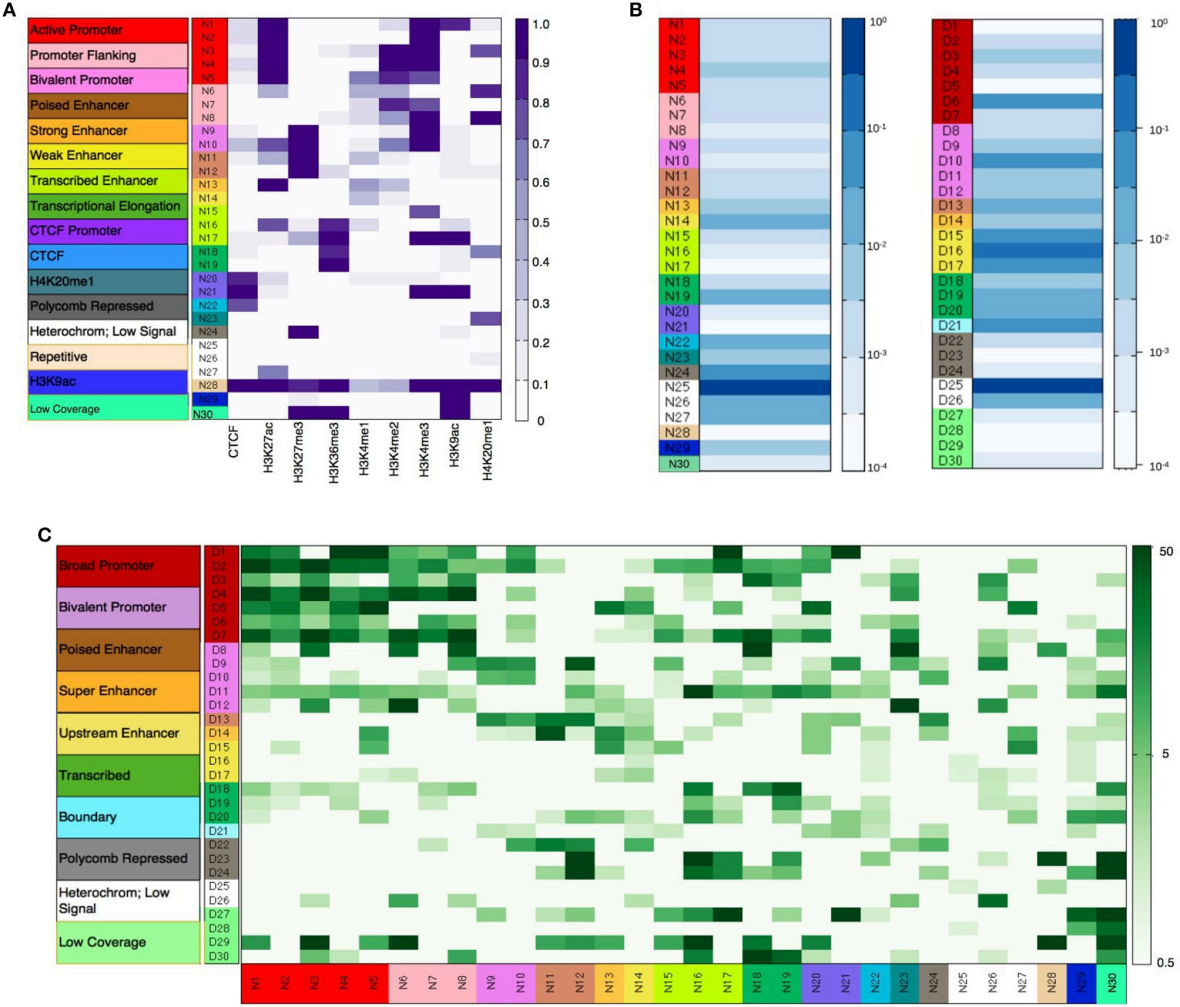
**(A)** Nucleosome level emission matrix generated by diHMM. Functional annotations of the nucleosome level states are shown in the color bar on the left. Scale varies linearly between 0 and 1. **(B)** Fractional genome coverage for nucleosome and domain level states. Scale varies logarithmically between 10^−4^ and 1. **(C)** Combined nucleosome-domain fold change obtained by diHMM. Functional annotation of the states are shown in the color bar on the left. Scale varies logarithmically between 0.5 and 50.

As described in the methods section, diHMM segments a genome into distinct chromatin states and outputs the states as regions within two bed files labeled by nucleosome and domain indexes (e.g., N1, N2…, and D1, D2…respectively). For the nucleosome level states, annotation of the chromatin states to functionally relevant categories was performed by using information from the emission probabilities of the nucleosome states (Figure 2A), fractional genome coverage (Figure 2B), relative enrichment in different genomic regions (Supplementary Figure S3), and distribution of nucleosome states around TSS (Supplementary Figure S4A). Similarly, by comparing the nucleosome-level fold enrichments in each domain level state and the distribution of the domain level states around TSS (Supplementary Figure S4B), the domain-level states were further grouped into different broader functional categories as shown in Figure 2C. Details of functional annotation of the nucleosome and domain-level states are presented in Section 3 of the Supplementary Document.

### 3.2. Chromatin State Preference of Individual Protein Binding and Gene Expression Regulation

**Figure 3:**
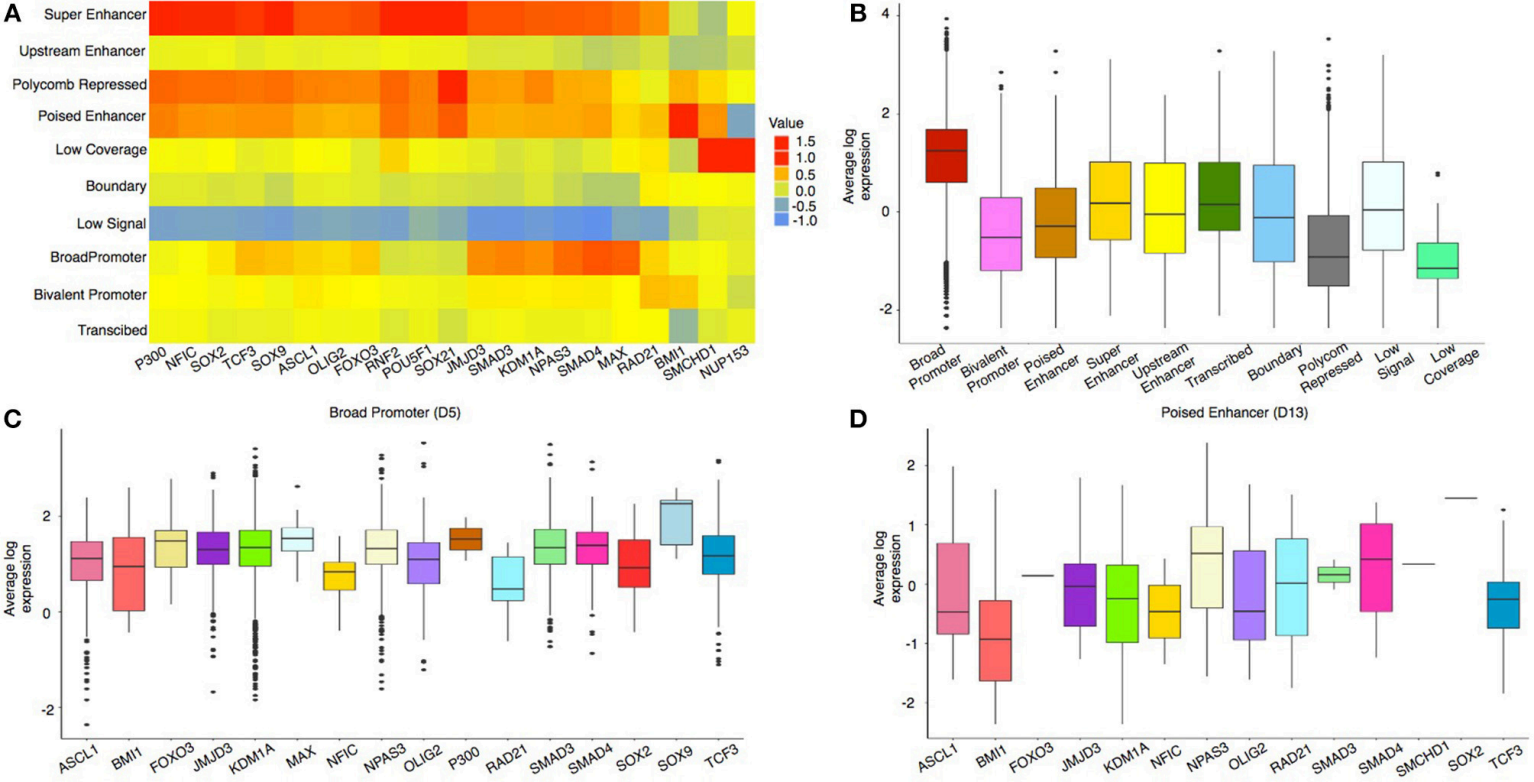
**(A)** Enrichment (in log scale) of TF peaks in different chromatin states showing binding preference of individual TFs. **(B)** Comparison of average TPM expression (in log scale) of proximal genes (± 2 kb from TSS) in different domain level chromatin states. Genes were mapped to the nucleosome-level states for the corresponding domain-level states. **(C)** Comparison of average TPM expression (in log scale) of proximal genes (± 2 kb from TSS) mapped to individual TFs in the Broad Promoter state and in **(D)** the Poised Enhancer state.

To analyze the distribution of protein-DNA binding sites in each chromatin state, we integrated ChIP-seq data with the chromatin state map of mouse neural stem cells (NSCs) (Figure 3A). For most proteins, the binding events occur in open chromatin regions, although some pioneer transcription factors have the ability to bind directly to condensed chromatin and recruit co-factors (Cuesta et al., 2007; Zaret and Carroll, 2011; Soufi et al., 2015). We observed, in both active and repressed states, enrichment of pioneer TFs as well as other proteins (that might have been recruited by the former). BMI1, which is known to bind to regions marked by both H3K27me3 and H3K4me3 (Bhattacharya et al., 2015), was found to be highly enriched in the Bivalent Promoter and Poised Enhancer states (Figure 3A). In addition, most TFs were found to be enriched in the Super Enhancer states except for RAD21, BMI1, SMCHD1, and NUP153. A similar observation was made by the authors in Mateo et al. (2015) where they showed that OLIG2, NFI family, SOX2, SOX9, TCF3, FOXO3, ASCL1, SOX21, and MAX were associated with active enhancer regions.

Next, to study the regulatory effect of histone marks on proximal genes, we compared the expression levels of genes (Transcripts Per Kilobase Million or TPM) with promoters located in different chromatin states. We observed that proximal genes in the Broad Promoter state had a higher median expression than proximal genes in the Polycomb Repressed or Low Coverage states (Figure 3B). To understand the influence of chromatin states on transcriptional regulation, we further grouped genes in each state based on the presence of binding sites of different proteins surrounding their TSSs. We observed that, for most proteins, the median expression of the genes in active states was higher than those in repressed states (Figures 3C,D and Supplementary Figure S8). Also, fewer proteins had binding sites in repressed states as compared to active states (In Figure 3C, there are 16 proteins whereas in Figure 3D, there are 14 proteins). Additional gene expression analysis for individual proteins is shown in Supplementary Figure S8.

### 3.3. Chromatin State and Preferential Clustering of Proteins

The distributions of ChIP-seq peaks across distinct chromatin states indicate that functionally relevant proteins may have similar binding patterns (Supplementary Figure S2). We determined the co-occupancy of proteins in a specific chromatin state through a nonparametric Bayesian clustering approach that identifies the combinatorial binding patterns of proteins (detailed description available in Supplementary Document). Each state at the domain level had multiple windows over different chromosomes across the genome. We observed that most windows are with very few peaks although the average domain-level window length ranged from 3.8 kb to over 450 kb. This prevented prediction of modules within a single domain window. To ensure that the unique properties of the domain-level states were preserved during clustering, we merged all windows of a single domain-level state (e.g., D1) across the entire genome and mapped the genome positions to a common interval [0, 50] on an imaginary real line. Adopting this approach for all domain level states eliminated the problem that different domains may have different sizes. Next, for each domain level state, the proposed algorithm used these mapped binding locations, computed individual binding intensity of each protein and clustered proteins having similar intensity patterns together to construct transcriptional regulatory modules. This process was repeated for each domain level state.

**Figure 4:**
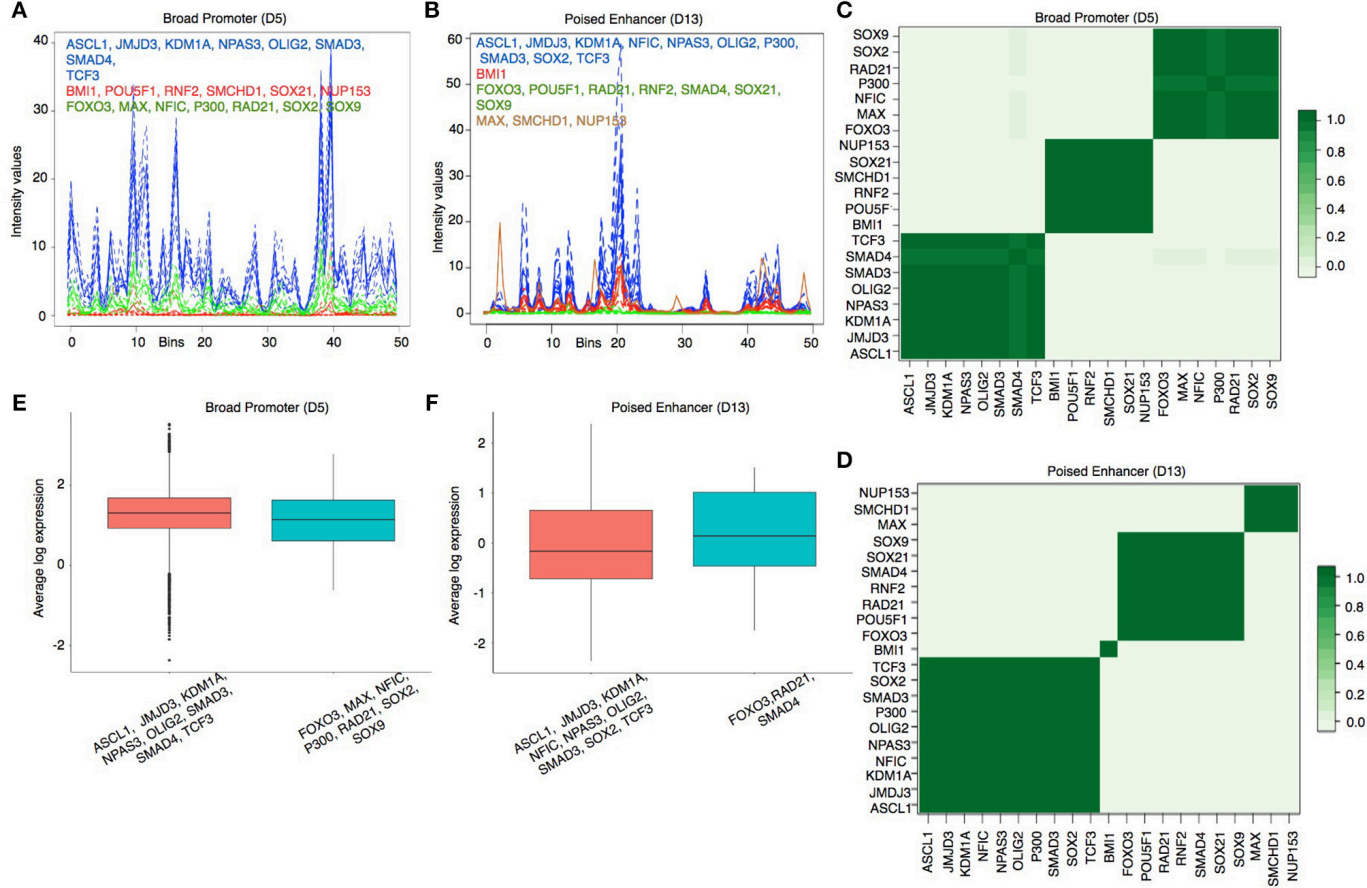
**(A,B)** Estimated cluster binding intensities along with the individual TF binding intensities in the Broad Promoter and Poised Enhancer states, respectively. In each figure, the estimated binding intensities of the individual TFs are shown in dotted lines and the estimated binding intensities of the clusters are shown in solid line. TFs in each cluster are shown in the same color as that of the cluster. The X axis represents the genomic locations mapped on the real line between 0 and 50. The Y axis represents the estimated binding intensities, both for the individual TFs and for the identified clusters. **(C,D)** Pairwise protein co-binding probabilities corresponding to **(A,B)** respectively. **(E,F)** Comparison of proximal gene expressions (TPM) regulated by the clusters in (a) and **(B)** respectively. Only those clusters having (1) multiple TFs and (2) proximal genes for at least two TFs are shown in the figure to explain the combinatorial regulation of gene expressions by multiple TFs.

To visualize the predicted regulatory modules in different chromatin states, we have shown the estimated binding intensities of the proteins and the corresponding clusters in Figures 4A,B and in Supplementary Figures S6, S7. We took a closer look at the clustering results in two contrasting states—Broad Promoter (Figure 4A) and Poised Enhancer (Figure 4B), and found noticeable differences in the binding intensity shape of both individual proteins and the predicted clusters between the two states. In addition, the set of co-factors for different proteins varied between the two states. BMI1 is known to bind to repressed and poised states (Bhattacharya et al., 2015) and was predicted as a single-protein cluster in the Poised Enhancer (Figure 4B) and Bivalent Promoter states (Supplementary Figure S6). In other states such as Broad Promoter, Super Enhancer, and Upstream Enhancer, BMI1 was predicted with RNF2, RAD21, or SMCHD1 (Supplementary Figures S6, S7). It is worth noting that both BMI1 and RNF2 are components of the Polycomb group multi-protein, whereas SMCHD1, a non-canonical member of the SMC super-family, is also known to be associated with transcriptional repression (Chen et al., 2015) and polycomb recruitment mechanisms (Gendrel et al., 2012). The proposed approach was able to cluster several other functionally relevant proteins that shared similar binding patterns, for example, JMJD3-SMAD3 (Figure 4) in most chromatin states (in Estarás et al., 2012, the authors found that JMJD3 is recruited to gene promoters by SMAD3 in neural stem cells and is essential to activate TGF-β-responsive genes), FOXO3-NFIC-SOX-TCF3 (Supplementary Figure S6) in Upstream Enhancer states (in Mateo et al., 2015), the authors showed interactions among NFI family, TCF3, SOX2, SOX9, and FOXO3. We have shown additional predicted protein-protein interactions in Supplementary Table S1.

To assess the strength of association between two co-binding proteins, we calculated a pairwise protein co-binding probability matrix from the posterior samples of the MCMC procedure Figures 4C,D). Each value in Figures 4C,D indicates the frequency of observing the corresponding two proteins in the same cluster out of the total 200 MCMC iterations. A high protein co-binding probability (indicated by darker color) provides stronger evidence of the existence of the protein pair in a cluster. We further performed a three-fold assessment on the robustness of the clustering algorithm explained in Supplementary Document Section 5.

We next examined the expression levels of proximal genes (Transcripts Per Kilobase Million or TPM) regulated by the predicted clusters in each state to understand transcriptional regulation by combinatorial binding of proteins in different chromatin states. We observed that the median expression level of the genes regulated by distinct clusters are close to each other in the Broad Promoter state (Figure 4E). On the contrary, the median expression level of the proximal genes combinatorially regulated by the FOXO3-RAD21-SMAD4 cluster in Poised Enhancer was higher than that of the genes combinatorially regulated by the other cluster (Figure 4F) (Similar behavior was observed in Bivalent Promoter, Upstream Enhancer and Boundary states shown in Supplementary Figure S9). These results show that gene expression could change due to combinatorial binding of proteins in different chromatin states.

### 3.4. Comparison of Results With Other Clustering Methods

We compared the clustering results of the proposed algorithm with K-means and CLARANS (Ng and Han, 2002). Instead of applying these two clustering methods directly on the binding locations of the proteins, we first estimated individual protein binding intensities and used these intensity matrices as inputs for clustering (we assumed each protein was in its own cluster). For both methods, we first obtained the optimal number of clusters using the NBclust package (Charrad et al., 2014). From the results in Table 1, we observe that for both methods, the number of optimal clusters was 2 for the two chromatin states. However, the cluster compositions that contain the regulatory TF modules are very similar to that of the proposed approach. Furthers comparisons are provided in Supplementary Table S5.

**Table 1:** Comparison of clustering results with other methods.

**Chromatin state**	**DPM-LGCP**	**K-means**	**CLARANS**
Broad Promoter (D5)	(1) ASCL1, JMJD3, KDM1A, NPAS3, OLIG2, SMAD3, SMAD4, TCF3; (2) BMI1, POU5F1, RNF2, SMCHD1, SOX21, NUP153; (3) FOXO3, MAX, NFIC, P300, RAD21, SOX2, SOX9	(1) ASLC1, JMJD3, KDM1A, NFIC, NPAS3, OLIG2, SMAD3, SMAD4, TCF3; (2) BMI1, FOXO3, MAX, P300, POU5F1, RAD21, RNF2, SMCHD1, SOX2, SOX21, SOX9, NUP153	(1) ASCL1, FOXO3, JMJD3, KDM1A, NFIC, NPAS3, OLIG2, RAD21, SMAD3, SMAD4, SOX2, SOX9; (2) BMI1, MAX, P300, POU5F1, RNF2, SMCHD1, SOX21, NUP153
Poised Enhancer (D13)	(1) ASCL1, JMJD3, KDM1A, NFIC, NPAS3, OLIG2, P300, SMAD3, SOX2, TCF3; (2) BMI1; (3) FOXO3, POU5F1, RAD21, RNF2, SMAD4, SOX21, SOX9, TCF3; (4) MAX, SMCHD1, NUP153	(1) ASCL1, JMJD3, KDM1A, NFIC, NPAS3, OLIG2, P300, SMAD3, SOX2, SOX9, TCF3; (2) BMI1, FOXO3, MAX, POU5F1, RAD21, RNF2, SMAD4, SMCHD1, SOX21, NUP153	(1) ASCL1, FOXO3, JMJD3, KDM1A, NFIC, NPAS3, OLIG2, P300, POU5F1, SMAD3, SMAD4, SOX2, SOX9, TCF3; (2) BMI1, MAX, RAD21, RNF2, SMCHD1, SOX21, NUP153

*For each method the clusters are preceded by the cluster number within parentheses. Further comparisons are shown in Supplementary Table S5*.

### 3.5. Protein-DNA Binding Motif Preferences in Chromatin States

**Figure 5:**
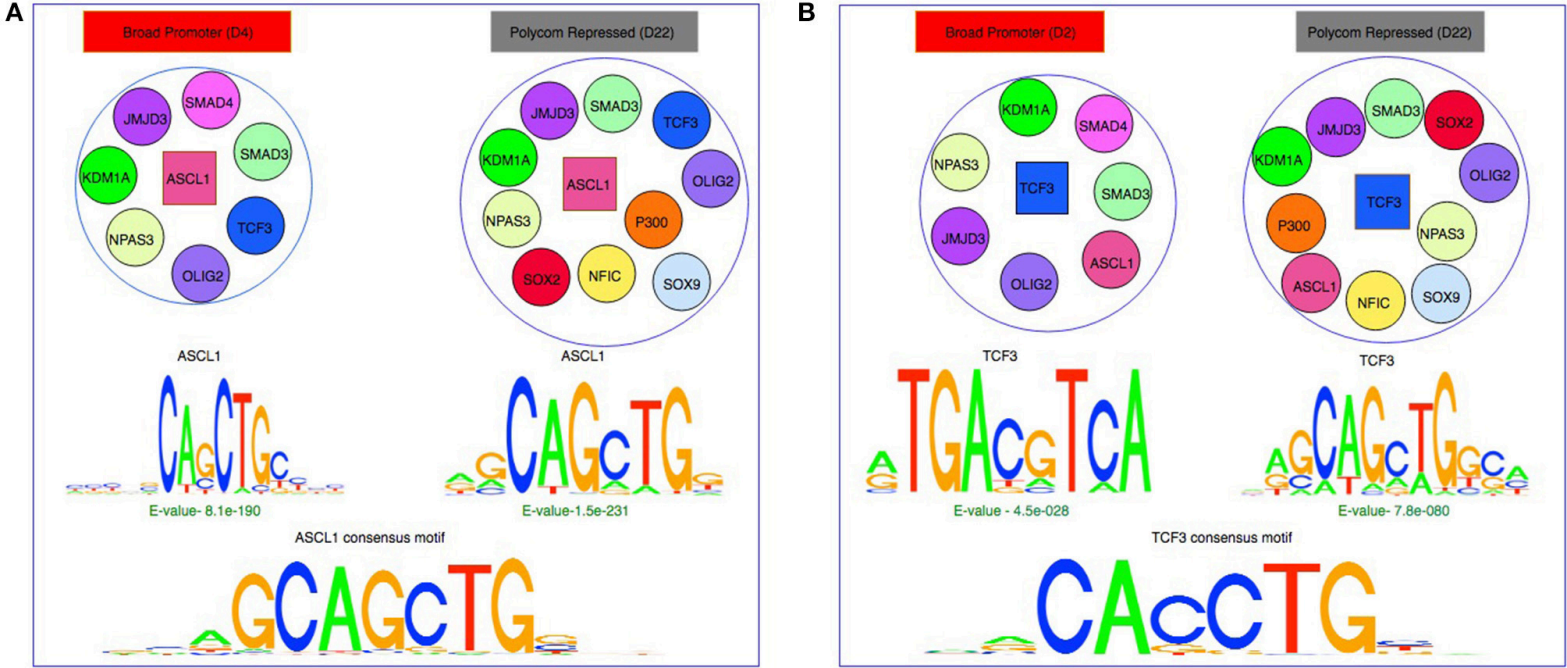
Effect of chromatin states and co-binding partner on binding motifs. **(A)** *De-novo* motifs obtained using MEME for ASCL1 are similar to the consensus motif in both Broad Promoter and Polycomb Repressed states although the co-factors of ASCL1 are different in the two states. **(B)** *De-novo* motifs obtained using MEME for TCF3 show differences in motifs between the two states with different co-factors. The motifs in active state resemble the β-catenin/TCF/LEF motif whereas the motifs in repressed state resemble the E-Box consensus motif.

It is known that local epigenetic states affect bindings of proteins to targets and protein-DNA binding may prevent or facilitate epigenetic changes on their binding sites (Blattler and Farnham, 2013; Xin and Rohs, 2018). A protein is known to bind to the DNA with different motifs depending on the presence of its co-binding partners (Bais et al., 2011). To examine the influence of chromatin states and co-binding partners on the binding sequences of a protein, we grouped ChIP-seq peaks for each protein overlapped with each chromatin state and analyzed the binding motifs of the protein in an active (Broad Promoter/Super Enhancer) and a repressed state (Poised Enhancer/Polycomb Repressed) (Figures 5A,B). We used the MEME suite (Bailey et al., 2009) to identify *de-novo* motif sequences and from the results we selected the motif that matched with the candidate protein's consensus motif or was known as a secondary motif. In both the HOMER (Heinz et al., 2010) or JASPAR (Mathelier et al., 2016) databases, no reference motif is documented for BMI1, KDM1A, JMJD3, NPAS3, NUP153, RNF2, RAD21, P300, and SMCHD1. For the remaining proteins with known motifs, we extracted genomic sequences from two different subsets of peaks overlapped with two contrasting chromatin states as mentioned before and determined the *de-novo* motifs.

Based on the MEME results, a protein's binding preferences may be broadly categorized into one of the three types: (1) *de-novo* sequences that closely matched the protein's consensus motif such as ASCL1 (Figure 5A), MAX, NFIC, FOXO3, and TFs from the SOX family. (2) *De-novo* sequences that either did not match with the consensus/secondary motifs or matched the consensus motif but were weakly enriched. It has been observed that the ATF/CREB motifs (“TGAYRTCA”) are often enriched in genes targeted by β-catenin/TCF/LEF (Lien et al., 2014; Taniue et al., 2016). For TCF3, we observed highly enriched *de-novo* sequences resembling its consensus motif in the repressed state (Figure 5B). However, in the active state we observed that the “TGACGTCA” pattern was highly enriched. This could imply that TCF3 might have been recruited by other co-factors resulting in indirect binding in that particular state. For OLIG2, both active and repressed chromatin states contained *de-novo* sequences resembling its consensus motif. However, these sequences were highly enriched in the repressed state and weakly enriched in the active state. The fact that the *E*-value of the *de-novo* sequences of OLIG2 was not significant in the active state might suggest indirect binding in the state, probably being governed by other factors. (3) *De-novo* sequences resembling the secondary motifs such the SMAD family. For SMAD4, we observed that sequences with ‘GCCGC' pattern were highly enriched in both active and repressed chromatin states, as reported previously in Hu et al. (2013) where the authors found that SMAD4 can bind to both methylated and un-methylated motifs of distinct sequences. Similarly, for SMAD3, we observed highly enriched sequences rich in “GC” content in both chromatin states, which have been reported as secondary SMAD3 motifs, often associated with known SMAD binding partners in TGF-β responses (Vidakovic et al., 2015). Interestingly, for POU5F1, we observed that the E-Box element “CANNTG” was significantly enriched in both active and repressed chromatin states. In Yin et al. (2017), the authors had also observed that the E-Box motif was significantly enriched with a *p*-value of 1e-6 in a POU5F1 ChIP-seq experiment of ES cell with Dnmt1, Dnmt3A and Dnmt3B triple knockout, whereas the consensus POU5F1 motif was weakly enriched with a *p*-value of 0.1. Detailed results are provided in Supplementary Table S3.

## 4. Discussion

Development of the semi-automated genome annotation tools has enabled genome segmentation and identification of distinct chromatin states at fine resolutions. In this study, we designed a two-step process to identify transcriptional regulatory modules within distinct chromatin states. First, we segmented the genome using the diHMM software. Second, we designed a novel nonparametric Bayesian clustering algorithm to identify clusters of co-binding proteins on the segmented genome. Existing work have adopted distance thresholds and empirical tests to define pairwise co-bound regions and context-dependent co-regulators (Ji et al., 2006; Chen et al., 2008; Orlov et al., 2009; Lee and Zhou, 2013). The statistically principled approach we proposed models protein-DNA binding site locations through inhomogeneous Poisson processes. It also employs a Dirichlet process prior to the random distribution of the latent log-intensity functions to facilitate clustering of the binding patterns. Such a nonparametric Bayesian clustering procedure is based on joint likelihood rather than pairwise protein-protein relationship and is flexible in capturing the intricate protein-DNA binding patterns in ChIP-seq data. This approach does not require pre-specified parameters such as window size, distance threshold, and number of clusters, and hence achieves improved robustness.

We applied the approach on ChIP-seq data for neural stem cells obtained from ChIP-Atlas, an integrated and comprehensive database rapidly gaining importance in cell replacement therapy. Despite the methodological advantages, this approach may have limitations in practical use. First, ChIP-seq can produce millions of short reads, which may result in varying strengths of signal intensities along the genome. In the current study, we did not consider the peak-height for different proteins but treated the center of each peak as a binary binding event along the genome. The overlook of the signal intensity effects may impact the modeling of protein binding patterns. Another possible limitation of our approach lies in handling the three dimensional structural information of the histone marks. This restricted our downstream gene expression analysis to gene promoters present in the Enhancer states. While not in scope of the current study, including such information may improve the accuracy of the model and enable the prediction of long distance Enhancer activity.

Nevertheless, we were able to establish several interesting findings. It has been known that protein-DNA binding sites are not randomly distributed but rather clustered together at enhancer or promoter regions. Hence, some specific proteins may team up to have a significant epigenetic impact on gene expression. In our study, transcriptional regulatory modules identified in different chromatin states revealed several known protein-protein interactions in neural stem cells, for example, SOX family and NF1 in the Enhancer states (Webb et al., 2013), MAX-FOXO3-OLIG2 in Upstream Enhancer (Mateo et al., 2015), and JMJD3-SMAD3 in most chromatin states (Estarás et al., 2012). These results suggest chromatin-state-specific protein-protein co-occupancy. In addition, diverse gene expression levels were observed through combinatorial regulation by the predicted transcriptional regulatory modules in different states. The uncovered links between gene expression and protein binding patterns on a genome-wide scale will enhance our understanding on how chromatin-state-specific regulatory network is assembled to coordinate tissue differentiation and cell specification.

An important issue in transcription regulation is to understand the binding specificity and affinity of a protein. A TF may have several thousands of DNA binding sites along the genome, which collectively can be represented as a motif—a consensus sequence demonstrating the nucleotide preferences at each position of the binding site. In this study, we observed that chromatin state can have an impact on the binding preferences of transcription factors and their co-activators (Jolma et al., 2015). For example, the *de-novo* sequences predicted for the some proteins resembled the consensus PWM across distinct chromatin states whereas for certain proteins such as SMAD family the sequences resembled secondary motifs in specific chromatin states. Further, we also noticed that the prediction of binding preferences might help the identification of indirect protein bindings when the *de-novo* sequences do not match the consensus PWM (Yin et al., 2017). In conclusion, we expect that our work will help understand the causality of chromatin state and combinatorial protein-DNA binding in regulating gene expression in neural stem cells.

## Author Contributions

HX conceived and designed the study. HZ and XW designed and implemented the clustering model. SB and MT designed computational experiments and performed data analyses. HX, WF, XW, and SB wrote the original draft. All authors read and approved the final manuscript.

### Conflict of Interest Statement

The authors declare that the research was conducted in the absence of any commercial or financial relationships that could be construed as a potential conflict of interest.
