# Supplementary material for: Identifying Transcriptional Regulatory Modules Among Different Chromatin States in Mouse Neural Stem Cells
Source: Front Genet. 2019 Jan 15;9:731. doi: 10.3389/fgene.2018.00731 (PMC6341026; doi:10.3389/fgene.2018.00731)
Supplement: Supplementary file 3 [file Data_Sheet_1.PDF]

Supplementary document

November 27, 2018

1 Dirichlet Process mixture of log Gaussian Cox processes

We use the Log Gaussian Cox Process (LGCP) to describe the binding pattern of a single TF. Let $S = \{s_1, ..., s_n\}$ denote the set of binding sites (BS) of the TF on a particular region of the DNA. Mathematically, we assume that $s_i \in D, i = 1, \ldots, n$ where D is a closed interval on the real line, i.e., $D \subset \mathbb{R}$. Further, we assume that S follows an inhomogeneous Poisson process with intensity function $\lambda(s), s \in D$. The likelihood of S can be written as

$$f(S|\lambda(s)) = \exp\left\{|D| - \int_D \lambda(s)ds\right\} \prod_{i=1}^{n} \lambda(s_i), \tag{1}$$

where $|D|$ represents the length of the interval D. In an LGCP, we assume that the intensity function $\lambda(s)$ follows a Gaussian process in log scale, i.e., $\lambda(s) = \exp\{z(s)\}$, and $z(s)$ is a Gaussian process a priori: $z(s) \sim GP(0, C_{\boldsymbol{\theta}})$. Here, $C_{\boldsymbol{\theta}}(.,.)$ denotes the covariance kernel of $z(s)$ so that $\mathrm{Cov}(z(s_l), z(s_m)) = C_{\boldsymbol{\theta}}(s_l, s_m)$ and $\boldsymbol{\theta}$ denotes parameters in the covariance kernel.

Let N be the total number of TFs. We denote the BS for the ith TF by $S_i = \{s_{i1}, ..., s_{in}\}, i = 1, ..., N$. We further assume that the N TFs form K clusters with K unknown. Existing approaches often require pre-specifying K, and perform clustering based on features extracted from S_i or its intensity estimation [1]. Such approaches can be sensitive to the ad-hoc choice of K and different feature extraction approaches adopted. In this paper, we propose a nonparametric Bayesian approach which allows us to perform clustering of $\{S_i\}$ without pre-specifying K or extracting features. The proposed approach is a Dirichlet Process Mixture of Log Gaussian Cox Process (DPM-LGCP) with the following setup:

$$S_i \mid \lambda_i(s) \sim \text{inhomo-Poisson}(\lambda_i(s)),\ s \in D,\ i = 1, \ldots, N;$$
$$\log(\lambda_i(s)) = z_i(s);$$
$$z_i(s) \sim G;$$
$$G \sim DP(m, G_0);$$
$$G_0 = GP(0, C_{\boldsymbol{\theta}});$$

where m is the precision parameter and G_0 is the base measure of the Dirichlet process. The introduction of the Dirichlet process mixture (DPM) to the LGCP model naturally facilitates clustering of N point processes. Unlike existing work [1], the DPM-LGCP approach does not require specifying a distance measure between two point processes.

An equivalent representation of the above DPM-LGCP model is based on the stick-breaking representation of the DPM model. Let $c_i \in \{1, 2, ...\}$ be a latent variable indicating the cluster assignment of the ith point process, i.e. $c_i = k$ means that process i comes from the kth cluster. It is assumed that point processes from the same cluster share a common intensity function. Let $\{\lambda_k^*\}$ denote the unique intensity functions. The stick-breaking representation of the DPM-LGCP model is written as

$$S_i \mid c_i, \lambda_{c_i}^*(s) \sim \text{inhomo-Poisson}(\lambda_{c_i}^*(s)),\quad i = 1, \ldots, N;$$
$$Pr\{c_i = k\} = \pi_k,\ k = 1, 2, \ldots;$$
$$\pi_k = V_k \prod_{l=1}^{k-1} (1 - V_l),\ V_k \sim \text{Beta}(1, m);$$

$$\lambda_k^*(s) = \exp\{z_k^*(s)\},\ z_k^*(s) \sim GP(0, C_{\boldsymbol{\theta}}).$$

With the model described above, approximate Bayesian inference can be performed using Integrated Nested Laplace Approximations (INLA) package [2]. In particular, the INLA package provides the approximated marginal likelihood which can be combined with the DPM sampling. Details of the approximation and the computational algorithm are provided in Section 1.1.

1.1 INLA approximation of the LGCP model

To review the modeling of a spatial point process using INLA, let us consider the LGCP model of a single point process S. INLA adopts an explicit link between the latent Gaussian process $z(s)$ and a discrete spatial Gaussian Markov random field $z = (z_1, ..., z_P)$ [3, 4, 5]. In particular, one assumes that the covariance kernel of $z(s)$ follows a Matérn covariance structure with

$$C_{\boldsymbol{\theta}}(x, y) = \frac{\sigma^2}{\Gamma(\nu)2^{\nu-1}}(\kappa|x-y|)^\nu K_\nu(\kappa|x-y|),$$

where $K_\nu(.)$ is the modified Bessel function of the second kind, $\nu > 0$ is the smoothing parameter, $\kappa > 0$ is the range parameter, and σ^2 is the marginal variance. With this assumption, one can show that $z(s)$ is the solution of the stochastic partial differential equation (SPDE):

$$(\kappa^2 - \Delta)^{\alpha/2} z(s) = W(s), \tag{2}$$

where $\alpha = \nu - 1/2$ is an integer (by default $\alpha = 2$ in the INLA package), $\Delta = \partial^2/\partial s^2$ is the Laplacian operator, and $W(s)$ is the white noise. The solution $z(s)$ of the SPDE can be approximated numerically through expanding $z(s)$ on a B-spline basis ϕ_j:

$$z(s) \approx \sum_{j=1}^{P} \phi_j(s) z_j, \tag{3}$$

where ϕ_j is chosen to be the piecewise linear basis constructed using B-spline of order 1 on the interval D. This expansion will transform the continuous co-variance of $z(s)$ to a discrete precision matrix Q of the B-spline basis coefficients $\boldsymbol{z} = (z_1, ..., z_P)^T$. Q will be approximated using the properties of Gaussian Markov Random Field (GMRF, note: GMRF is defined discretely), which takes advantages of a local correlation structure in Q to facilitate fast computation. Through B-spline approximation, an explicit link is constructed between a continuous Gaussian process $z(s)$ and a discretely defined GMRF $\boldsymbol{z}$.

The implementation in INLA contains several steps. First, we construct a regular grid $\boldsymbol{t} = (t_1, ..., t_P)$ on D and construct a 1-D B-spline of order 1 on the grid. Under this construction, $\phi_j(t)$ is a piecewise linear base function that takes value 1 at node t_j and zero on the rest of the grid points. Accordingly,

$$\lambda(s) = \exp\{z(s)\} \approx \exp\{\sum_{j=1}^{P} \phi_j(s) z_j\}.$$

With the basis approximation, we can further approximate the integral in Equation (1) by

$$\int_D ds\ \lambda(s) \approx \sum_{j=1}^{P} \lambda(t_j)\tilde{\alpha}_j \approx \sum_{j=1}^{P} \exp\{\sum_{j=1}^{P} \phi_k(t_j) z_k\}\tilde{\alpha}_j.$$

Here $\tilde{\alpha}_j$ is the weight for numerical integration which takes the value $|D_j|$ where D_j is the support of $\phi_j(.)$. The exponential part of Equation (1) can then be approximated by

$$\exp\{|D| - \int_D ds\lambda(s)\} \approx \exp\{|D| - \sum_{j=1}^{P} \tilde{\alpha}_j \exp\{\sum_{j=1}^{P} \phi_k(t_j) z_k\}\} = \exp\{|D| - \tilde{\alpha}^T \exp(\boldsymbol{A_1 z})\},$$

where $\tilde{\alpha} = (\tilde{\alpha}_1, \ldots, \tilde{\alpha}_P)^T$ and $[A_1]_{jk} = \phi_k(t_j), j = 1, \ldots, P,\ k = 1, \ldots, P$. The product part of Equation (1) can be approximated by

$$\prod_{i=1}^{n} \lambda(s_i) \approx \prod_{i=1}^{n} \exp\{\sum_{j=1}^{P} \phi_j(s_i) z_j\} = \prod_{i=1}^{n} \exp\{\boldsymbol{A_2 z}\},$$

where $[\boldsymbol{A}_2]_{ij} = \phi_j(s_i), i = 1, \ldots, n, j = 1, \ldots, P$. Therefore, the log likelihood based on Equation (1) is approximated by

$$\log f(S \mid \lambda(s)) \approx |D| - \tilde{\alpha}^T \exp(\boldsymbol{A_1 z}) + \boldsymbol{1}^T \boldsymbol{A_2 z}. \tag{4}$$

Rewriting $\boldsymbol{\eta} = \exp(\boldsymbol{z^T A_1^T}, \boldsymbol{z^T A_2^T})$, $\boldsymbol{\alpha} = (\tilde{\boldsymbol{\alpha}}^T, \boldsymbol{0}_{n\times 1}^T)^T$ and constructing pseudo-observation $\boldsymbol{y} = (\boldsymbol{0}_{p\times 1}^T, \boldsymbol{1}_{n\times 1}^T)^T$, the LGCP likelihood in Equation (1) can be approximated using a Poisson density

$$f(\boldsymbol{y}|\boldsymbol{z}) \approx C \prod_{i=1}^{n+P} (\eta_i)^{y_i} \exp\{-\alpha_i \eta_i\},$$

which are the product of conditionally independent Poisson random variables with mean $\alpha_i \eta_i$ and observation y_i. INLA approximates the posterior distribution of $\pi(\boldsymbol{z}|\boldsymbol{y})$ using numerical integration:

$$\tilde{\pi}(z_i|\boldsymbol{y}) = \int \tilde{\pi}(z_i|\boldsymbol{\theta}, \boldsymbol{y})\tilde{\pi}(\boldsymbol{\theta}|\boldsymbol{y})d\boldsymbol{\theta},$$

The marginal posterior $\tilde{\pi}(\boldsymbol{\theta}|\boldsymbol{y})$ is an approximation to $\pi(\boldsymbol{\theta}|\boldsymbol{y})$ using

$$\tilde{\pi}(\boldsymbol{\theta}|\boldsymbol{y}) \propto \frac{\pi(\boldsymbol{z}, \boldsymbol{\theta}, \boldsymbol{y})}{\tilde{\pi}_G(\boldsymbol{z}|\theta, y)}\Big|_{\boldsymbol{z}=\boldsymbol{z}^*(\boldsymbol{\theta})},$$

where $\tilde{\pi}_G(\boldsymbol{z}|\boldsymbol{\theta}, \boldsymbol{y})$ is the Gaussian approximation of the full conditional of $\boldsymbol{z}$, and $\boldsymbol{z}^*(\boldsymbol{\theta})$ is the mode of the full conditional for $\boldsymbol{z}$ at a given $\boldsymbol{\theta}$. The marginal like likelihood $\pi(\boldsymbol{y})$ can be approximated by

$$\tilde{\pi}(\boldsymbol{y}) = \int \frac{\pi(\boldsymbol{\theta}, \boldsymbol{z}, \boldsymbol{y})}{\tilde{\pi}_G(\boldsymbol{z}|\boldsymbol{\theta}, \boldsymbol{y})}\Big|_{\boldsymbol{z}=\boldsymbol{z}^*(\boldsymbol{\theta})} d\boldsymbol{\theta}$$

using numerical integration. The marginal likelihood of S is denoted as $H(S) \approx \tilde{\pi}(\boldsymbol{y})$ in the DPM algorithm below.

1.2 Algorithm for posterior inference

Using Gibbs sampler, posterior inference of DPM-LGCP can be performed by the following algorithm.

Algorithm:

Step 0 Initialize the cluster assignment $c = (c_1, \ldots, c_N)$.

Step 1 For $i = 1 : N$, do the following: Exclude c_i from c. Denote the resulting number of clusters as K^-, the cluster sizes as $\{n_k^-\}$. Sample c_i from the following distribution:

$$(c_i \mid c_{-i}, \{S_i\}) = \begin{cases} k & \text{with prob.} \propto n_k^- H(S_i|\{S_l, c_l = k, l \neq i\}),\ k = 1, ..., K^- \\ K^- + 1 & \text{with prob.} \propto mH(S_i) \end{cases},$$

end For.

Repeat Step 1 for a pre-specified number of iterations.

Here, $H(S_i|\{S_l, c_l = k, l \neq i\}) = H(S_i, \{S_l, c_l = k, l \neq i\})/H(S_l, c_l = k, l \neq i)$, and all components are calculated using INLA.

2 Case studies

2.1 Simulated data

Three 1-D inhomogeneous Poisson processes with Log-Gaussian intensity were generated. Next, binding site locations of 20 transcription factors were simulated by drawing independent samples from these inhomogeneous Poisson processes. In Supplementary Figure S1, the solid curves denote the true intensities of the three processes.

Using the simulated binding site locations, we applied the clustering algorithm. The TFs were randomly assigned according to an initial cluster setting. We obtained the estimated clustering results after ten MCMC iterations, as shown in the summary below –

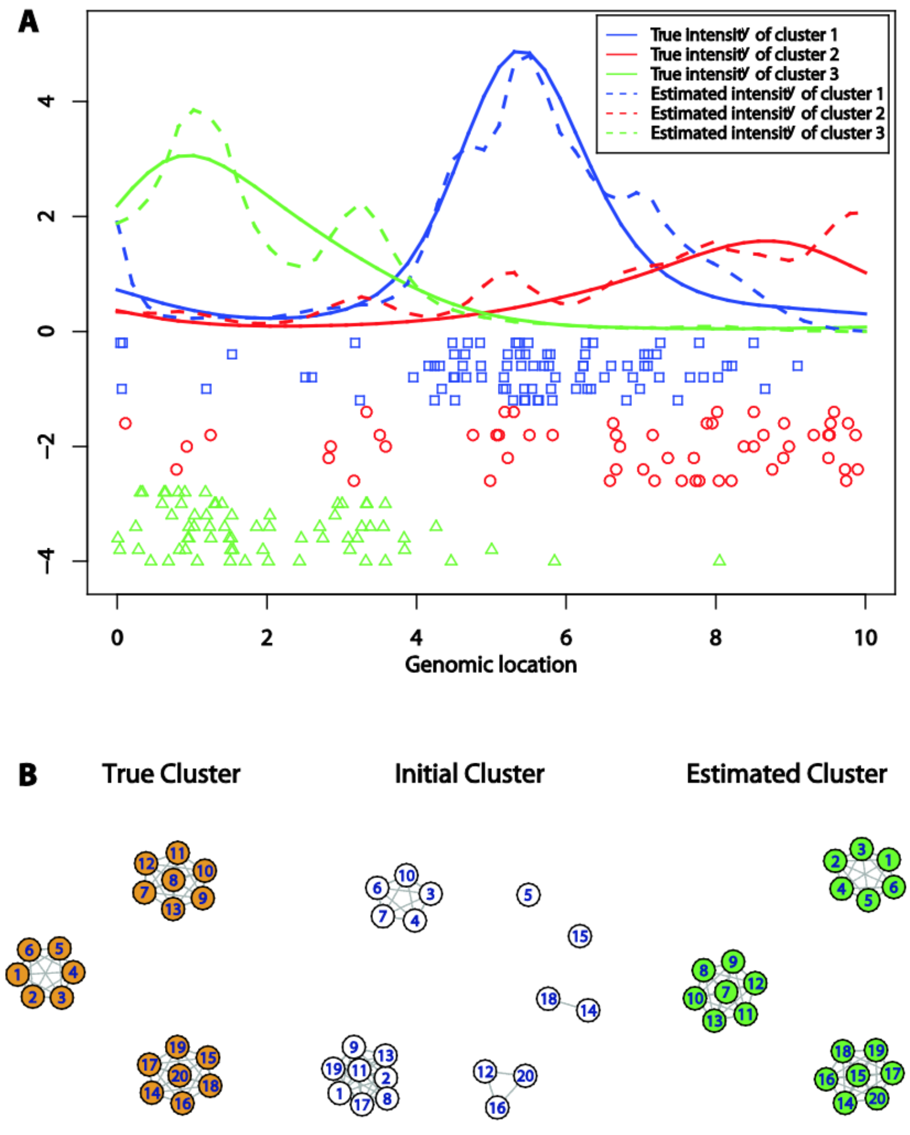

Supplementary Figure S1: Application of the clustering algorithm on simulated data. (A) True and Estimated binding intensities of twenty transcription factors in three clusters (B) Illustration of the clustering result obtained in (A).

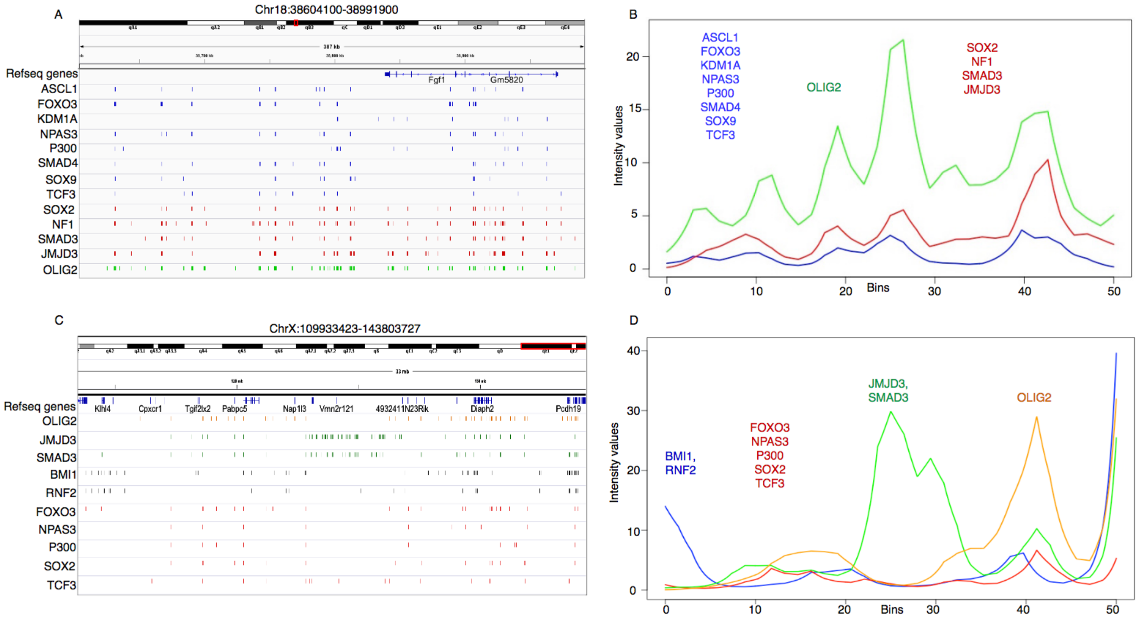

Supplementary Figure S2: Application of the clustering algorithm on real data. (A) Distribution of ChIP-seq peaks visualized in IGV window and (B) the corresponding estimated binding intensities of the clusters. The proteins in each cluster are shown with the same color.

1. initial error rate: 0.3947
2. estimation error rate: 0
3. overall true marginal likelihood: 76.7315
4. overall estimated marginal likelihood: 76.73146
5. true marginal likelihood per cluster: 27.2765, 14.9562, 34.4986
6. estimated marginal likelihood per cluster: 27.2765, 14.9562, 34.4986

2.2 Real data

An example of clustering on real data is shown in Supplementary Figure S2A,B. In the left panels, we visualize the ChIP-seq peak distribution of proteins obtained from ChIP-Atlas, in a specific genomic region using IGV software. The proposed algorithm uses these genome locations as input, re-scales them to an imaginary real line (from 0 to 50) and estimates the binding intensity of each TF using an inhomogeneous Poisson point process. Finally, proteins sharing similar intensity patterns are clustered together. In the right panels, the estimated cluster intensities are shown. The values on the Y-axis represent the magnitude of the intensity function, and the X-axis represents the bins of mapped genome locations on the real line. Proteins belonging to different clusters are marked in the same color as that of the cluster.

3 Chromatin state annotation by diHMM on real data from mouse neural stem cells

3.1 Datasets

ChIP-seq data used in this study were obtained from the ChIP-Atlas (http://chip-atlas.org) databases for mouse neural stem cells. We included 21 proteins in this study. Among these proteins, P300 is a co-activator, RAD21 is a sub-unit of cohesin component, SMCHD1 is a non-canonical member of the SMC protein family, NUP153 is one of the building blocks of the nuclear pore complex, JMJD3 is a lysine-specific demethylase that demethylates H3K27me2 or H3K27me3, KDM1A is also a histone demethylase that demethylates both H3K4me and H3K9me, RNF2

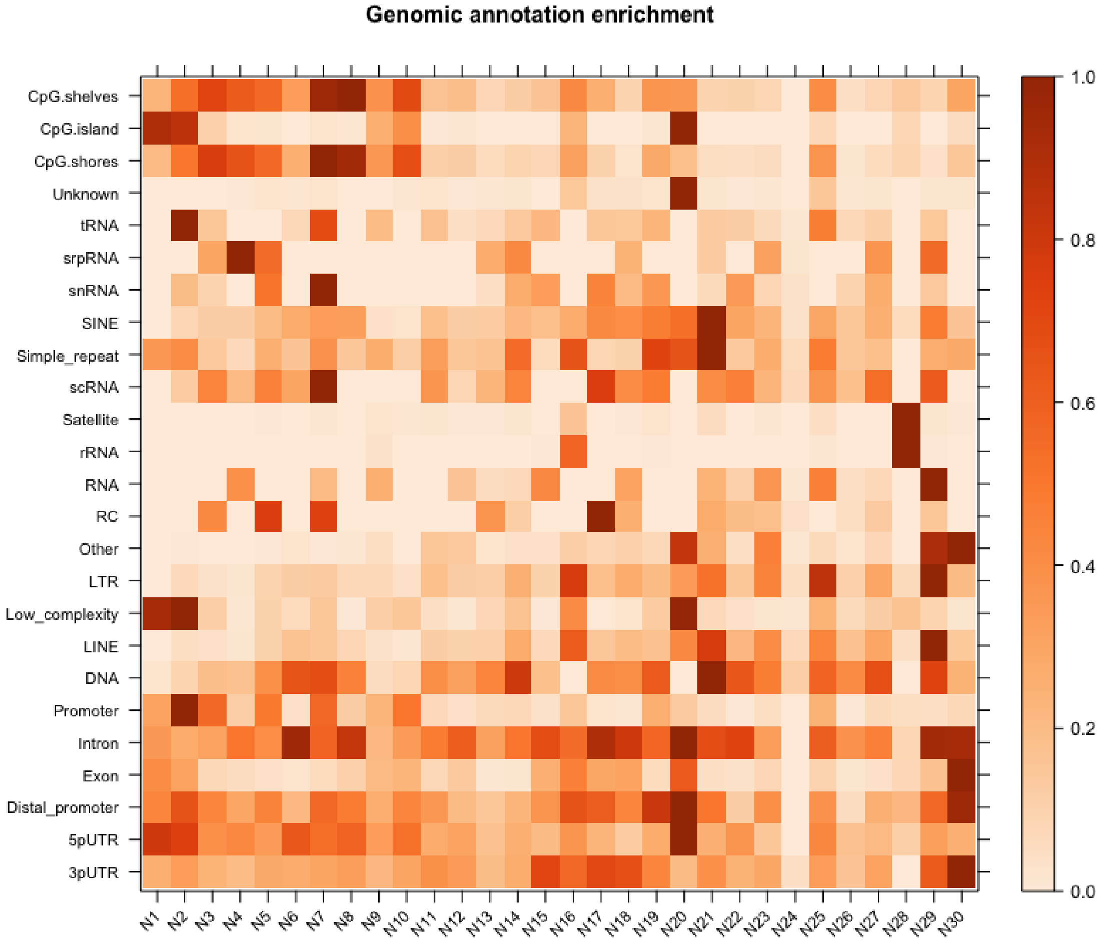

Supplementary Figure S3: Nucleosome states genome annotation enrichment.

which a E3 ubiquitin-protein ligase that mediates monoubiquitination of H2AK119Ub, BMI1 is a ring finger protein and a major component of the polycomb group complex 1 (PRC1), and SMAD3 and SMAD4 are signal transducers and transcriptional modulators mediating multiple signaling pathways and several other transcription factors such as OLIG2, FOXO3, ASCL1, MAX, NFIC, TCF3, SOX2, SOX21, SOX9, POU5F1, many of which are known to play key role in neural stem cells [6, 7]. Three major pre-processing steps were conducted, including alignment to reference genome using Bowtie2 [8] with default mode, binarization from SAM to BAM format using SAMtools [9] version 0.1.19, and peak calling using MACS2 [10] version 2.1.0 to obtain binding regions of the proteins. Gene expression data were downloaded from GSE70872 (untreated samples). Details of the datasets are provided in Supplementary Table S2.

3.2 Genome annotation enrichment

We downloaded CpG island sites, Refseq transcripts and Repeats for mm10 genome from UCSC database to calculate the fraction of genomic annotation for each nucleosome-level state (Supplementary Figure S3). We noticed that the Active Promoter states were enriched in the 5pUTR regions, while the Transcriptional Elongation states were enriched in the Intron regions. In addition, the Repetitive state was highly enriched in the Satellite regions.

3.3 Relative enrichment around TSS

We calculated the relative enrichment of the nucleosome level states with a maximum of +/- 4kb and domain level states with a maximum of +/- 80kb around transcription start sites (TSS). From the spatial distribution of the nucleosome level and domain level states (Supplementary

Figure S4), we observed that the Active Promoters, Bivalent Promoters at nucleosome level and Broad Promoters at domain level are enriched around the TSS. On the other hand, Trancriptional Elongation at nucleosome level and Upstream Enhancer at domain level are enriched away from the TSS. Repetitive and Low Signal states are enriched sporadically spanning on either side of the TSS.

3.4 State sizes

From the nucleosome and domain state sizes (Supplementary Figure S5), we observed that the Upstream Enhancer and Low Signal states at domain level have wider state sizes compared to the Broad Promoter and Super Enhancer states. The Low Signal states not only have larger fractional genome coverage at both nucleosome and domain levels (main text, Figure 2B) but also have in general larger state sizes. The Low Coverage states, on the other hand, have very small state sizes. Some of the Broad Promoter states also have smaller state sizes.

3.5 Functional annotation of nucleosome and domain level states

Nucleosome level states: Active Promoters (N1-N5: enriched in H3K27ac, H3K4me3, H3K4me2, and enriched around TSS), Promoter Flanking states (N6 to N8: enriched in H3K27ac, H3K4me3, and H3K4me2 flanking TSS), Bivalent Promoter (N9 and N10: enriched in H3K27me3 and H3K4me2 or H3K4me3), Poised Enhancer (N11 and N12: strong enrichment in H3K27me3, and weak enrichment in H3K4me1), Strong Enhancer (N13: enriched in H3K27ac and H3K4me1), Weak Enhancer (N14: enriched in H3K4me1), Transcribed Enhancer (N15-N17: enriched in H3K36me3 and sometimes in H3K4me3), Transcriptional Elongation (N18 and N19: enriched in H3K36me3), CTCF promoter (N20 and N21: enriched in CTCF and enriched around TSS), CTCF (N22: enriched in CTCF), H4K20me1 (N23: enriched only in H4K20me1), Polycomb Repressed (N24: enriched only in H3K27me3), Heterochrom/Low Signal (N25-N27: low enrichment in almost all marks and spanned over a large proportion of the genome, main text, Figure 2A,B), Repetitive (N28: enriched in multiple marks and in Satellite regions, Supplementary Figure S3), H3K9ac (N29: enriched only in H3K9ac), and Low Coverage (N30: low fractional genome coverage, main text, Figure 2B).

Domain level states: Broad Promoter (D1 to D7: enriched in active promoter and promoter flanking states), Bivalent Promoter (D8-D12: enriched in bivalent promoter, poised enhancer, and polycomb repressed states), Poised Enhancer (D13: enriched in poised enhancer), Super Enhancer (D14: enriched in active enhancer), Upstream Enhancer (D15-D17: enriched in weak enhancer and upstream from TSS), Transcribed (D18-D20: enriched in the transcribed elongation state and distributed over a broad region from TSS), Boundary (D21: enriched in CTCF domain), Polycomb Repressed (D22-D24: enriched in Poised Enhancer and Polycomb Repressed), Low Signal (D25-D26: not enriched in any state), and Low Coverage (D27-D30: low fractional genome coverage).

In addition, we observed that at both nucleosome and domain level the Low Signal state had the largest state sizes while the Low Coverage state had the smallest state sizes (Supplementary Figure S5).

4 Analysis of predicted clusters on mouse neural stem cells

4.1 Functional enrichment of proteins within chromatin states

We calculated the enrichment of each protein within each chromatin state to analyze positional preference of protein-DNA bindings. Enrichment of a particular protein in a particular chromatin state is calculated as $(m/n)/(M/N)$ where m is the number of peaks of the protein in that state, n is the total number of peaks of the protein in all states, M is the size of the state and N is the total size of all states. Functional enrichment analysis allows us to identify specific chromatin states that are highly enriched in protein-DNA bindings [11] and also specific states that are depleted in bindings, and thus provides an insight into genome wide binding preferences of proteins governed by different histone marks.

4.2 Gene expression and motif analysis

Gene expression level in a cell determines cell function and the initiation of transcription is the prime control point for gene expression regulation [12]. In this study, we compared the overall

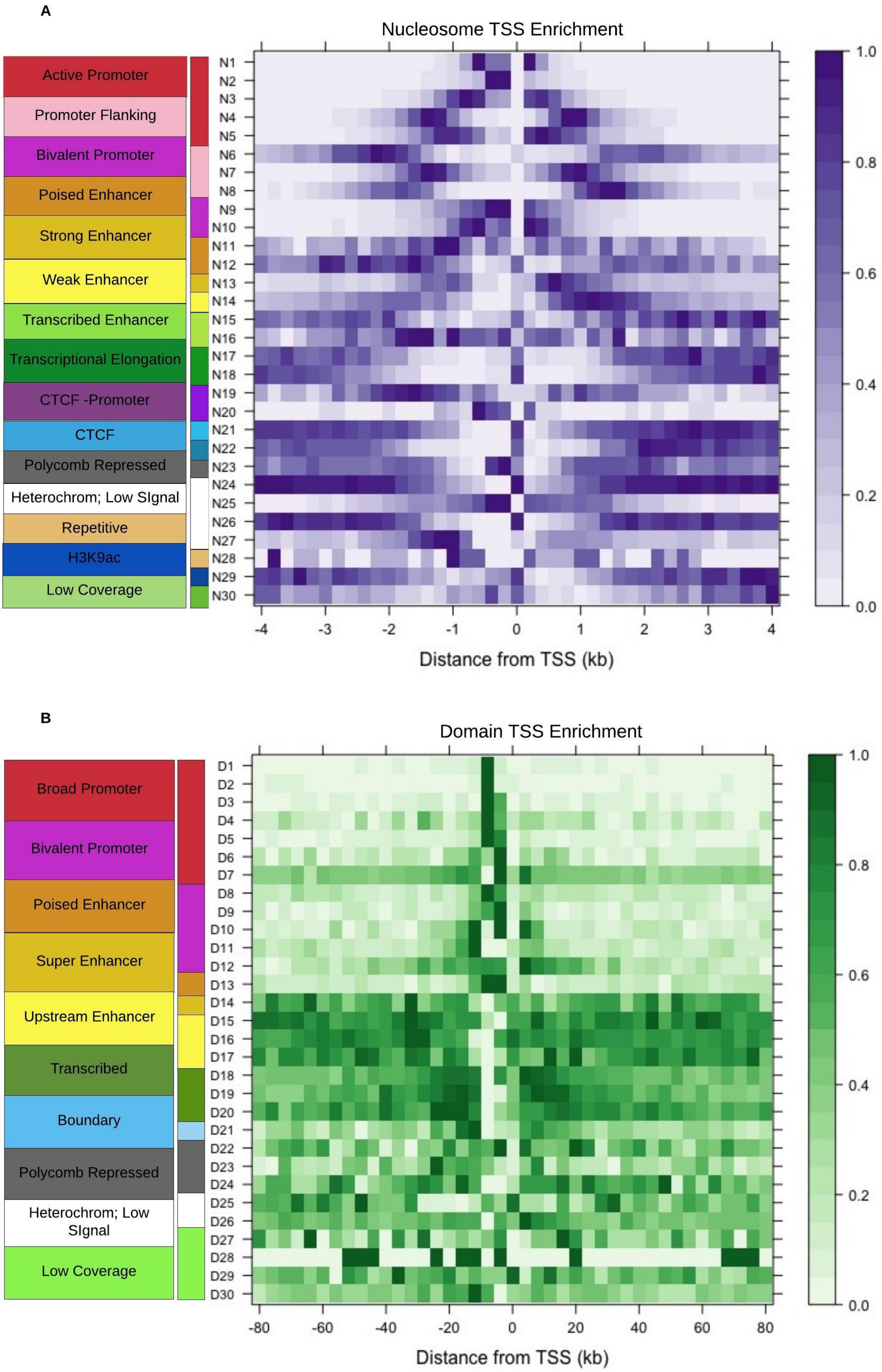

Supplementary Figure S4: Nucleosome and domain level state distribution around TSS.

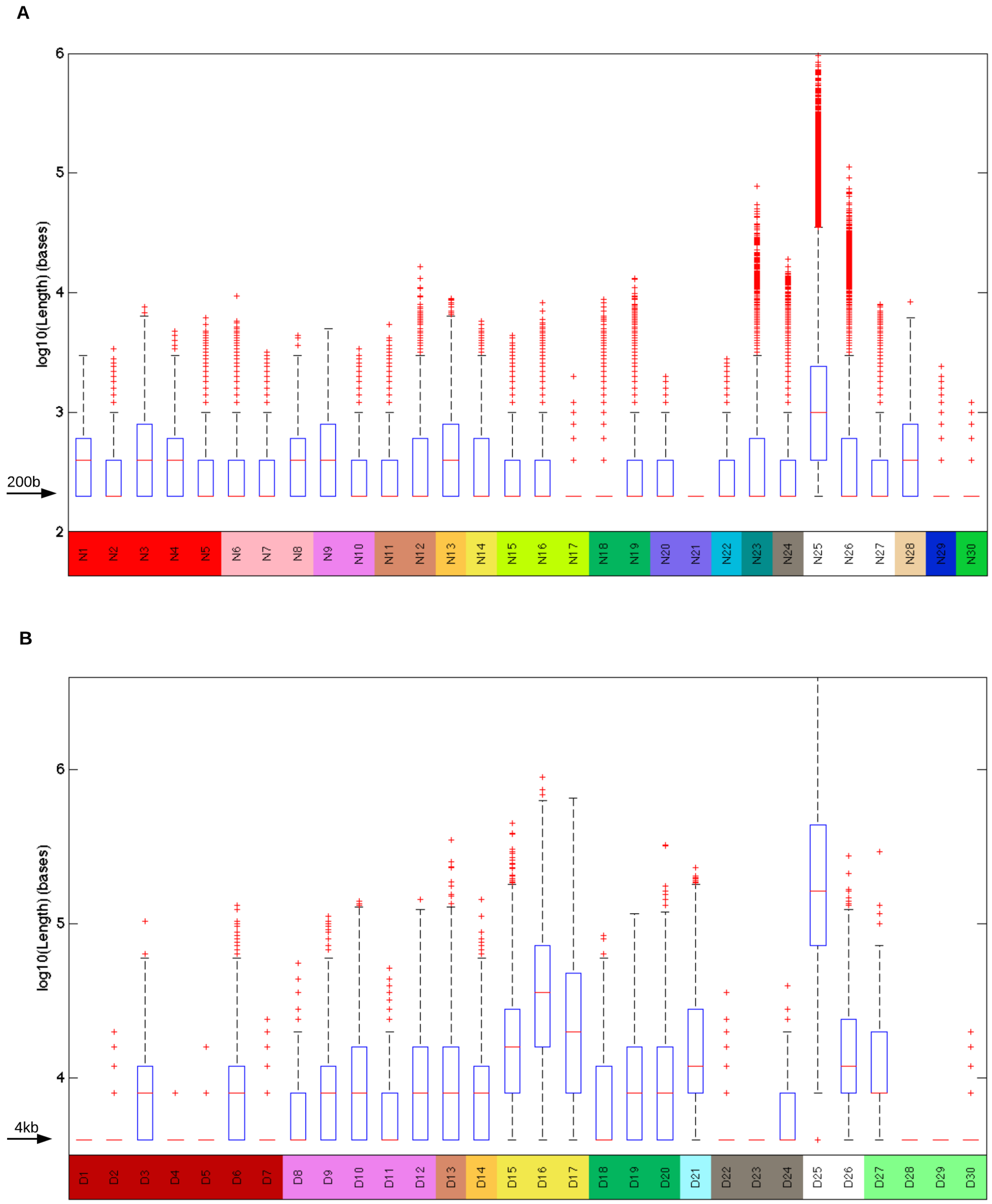

Supplementary Figure S5: Quartile box plots of $\log_{10}$ of all nucleosome-level (a), or domain-level (b) state sizes. Box plot whiskers extend to 1.5 times the interquartile range.

expression level of genes among different chromatin states. We also compared the regulatory effect of individual protein and the predicted modules on genes within each chromatin state. RSEM [13] was used to estimate gene expression (Transcripts Per Kilobase Million or TPM). Each gene had three expression values obtained from triplicates. For comparison of gene expression among chromatin states, genes were selected by proximity (+/- 2kb from TSS) to nucleosome-level state windows for the corresponding domain-level state windows. For comparison of gene expression regulated by different proteins within a specific chromatin state, genes were selected by proximity (+/- 2kb from TSS) to ChIP-seq peaks. We averaged the expression levels of each gene over replicates and then applied log transformation to the average expression levels. We also studied

the effect of chromatin states on the binding motifs [14] since the nucleotide sequences within protein-DNA binding sites are critical factors for recruiting both the primary protein and its cofactors. We used the MEME [15] software to discover de-novo motif sequences of proteins and compared those sequences to the consensus motifs from the HOMER [16] motif library.

4.3 Estimated clusters in chromatin states

Supplementary Figures S6 and S7 show the estimated binding intensities in the 28 chromatin states. In each figure the dotted lines represent the binding intensities of the individual proteins and the solid lines represent the estimated intensities of the identified clusters. The corresponding proteins in each cluster are shown in the same color as that of the cluster.

4.4 Known protein-protein interactions predicted by the algorithm

We have shown some of the known interactions predicted by the algorithm in Table S1. The first column shows co-occupancy of the proteins predicted by the algorithm together with the states where the co-occupancy was observed. The second column provides references that also had reported the predicted co-occupancies.

4.5 Comparison of proximal gene expression for individual proteins

Gene transcription start sites were extended by +/- 2kb. Next, genes were mapped by proximity to the binding peaks in each of the 30 domain-level states. It was observed that, in every state there were some proteins with no binding locations near proximal gene promoters (Supplementary Figure S8). Additionally, the Broad Promoter, Upstream Enhancer and Transcribed states had more binding sites near proximal genes than the repressed states such as Polycomb Repressed or Poised Enhancer.

Based on the clustering results, we grouped proteins predicted to share the same cluster and compared the gene expression levels jointly regulated by co-binding proteins among different clusters (Supplementary Figure S9).

5 Robustness of the proposed clustering technique

5.1 Effect of varying domain level state sizes

On a diHMM domain, we repeatedly sampled 1000 windows and applied our clustering algorithm on these windows. To assess the consistency of the results, we checked the error rates computed from the difference of the estimated clusters on the entire domain and the estimated clusters on every 1000 windows. To compute the error rate, we first define a protein-protein pair binary matrix with size N by N where N is the number of proteins. A '1' in the (i, j)th entry of the matrix indicates that protein i and protein j are in the same cluster and '0' otherwise. Next, we take the upper triangular half of this pair matrix (it is symmetric) and convert it to a vector of length $\binom{N}{2}$. Such a vector is obtained for the clustering result of each sub-sample and for the ground truth, and the error rate is reported by comparing the vectors obtained from sub-samples to that from the ground truth. We observed that the average error rate of misclassifying an edge was 0.1, which suggests that the clustering results are pretty robust to varying domain level state sizes. Supplementary Figure S10 shows the estimated intensity curves of the identified clusters in 10 such sub-samples, each with 1,000 randomly selected windows in a Broad Promoter domain (D5).

5.2 Effect of varying number of initial clusters

Like any unsupervised clustering algorithm, the proposed clustering process starts with a random initial cluster setting. In order to test whether the initial number of clusters affects the final results on a domain, we varied the initial number of clusters provided to the algorithm and checked the clustering results under these different settings. Since the total number of proteins used in the study is 21, we set the initial number of clusters to 2 and then gradually increased it to 21 (i.e., each protein forms a different cluster). We observed that the clustering results were invariant to the initial number of clusters provided as prior to the nonparametric Bayesian clustering algorithm.

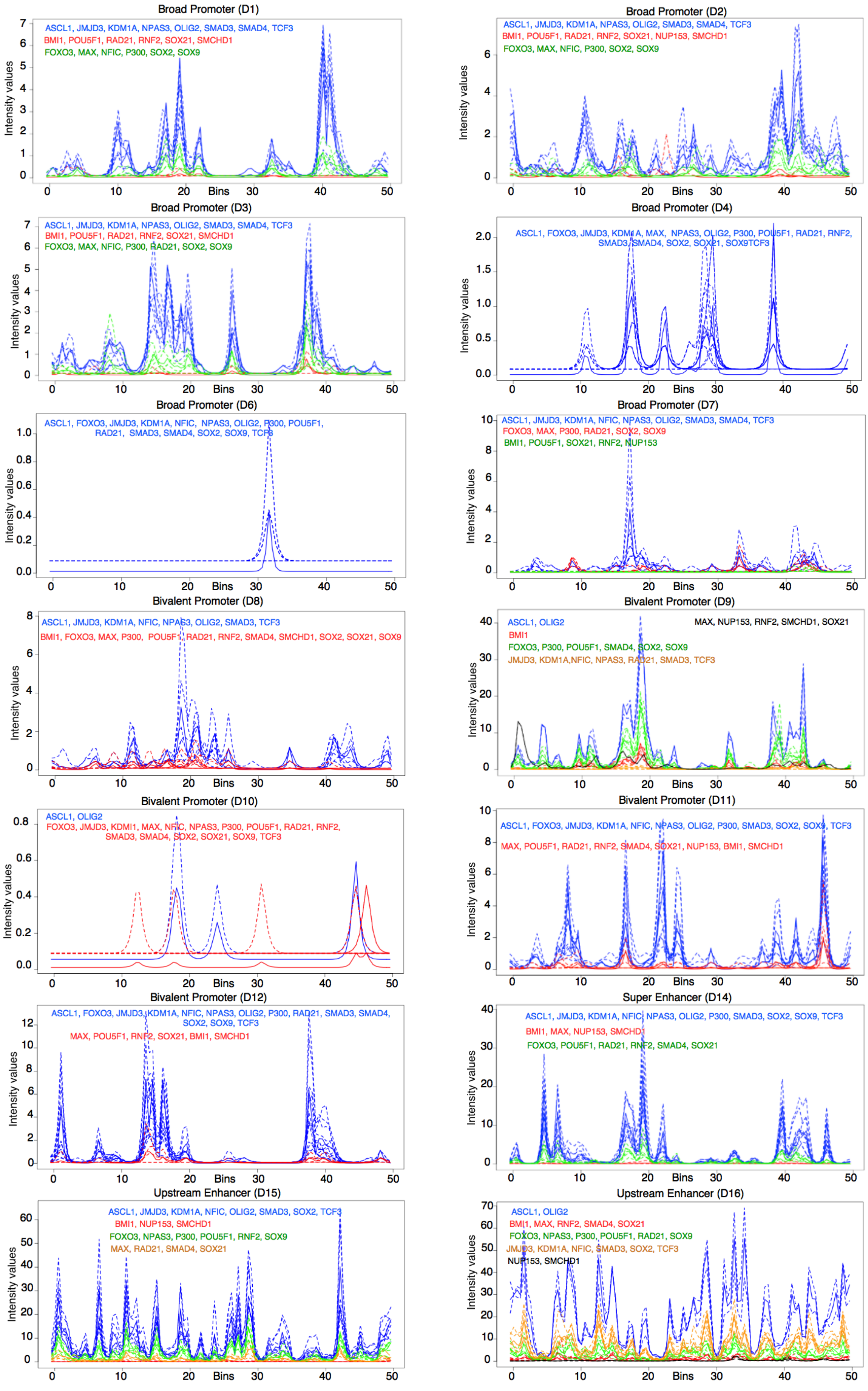

Supplementary Figure S6: Estimated cluster intensities in different chromatin states.

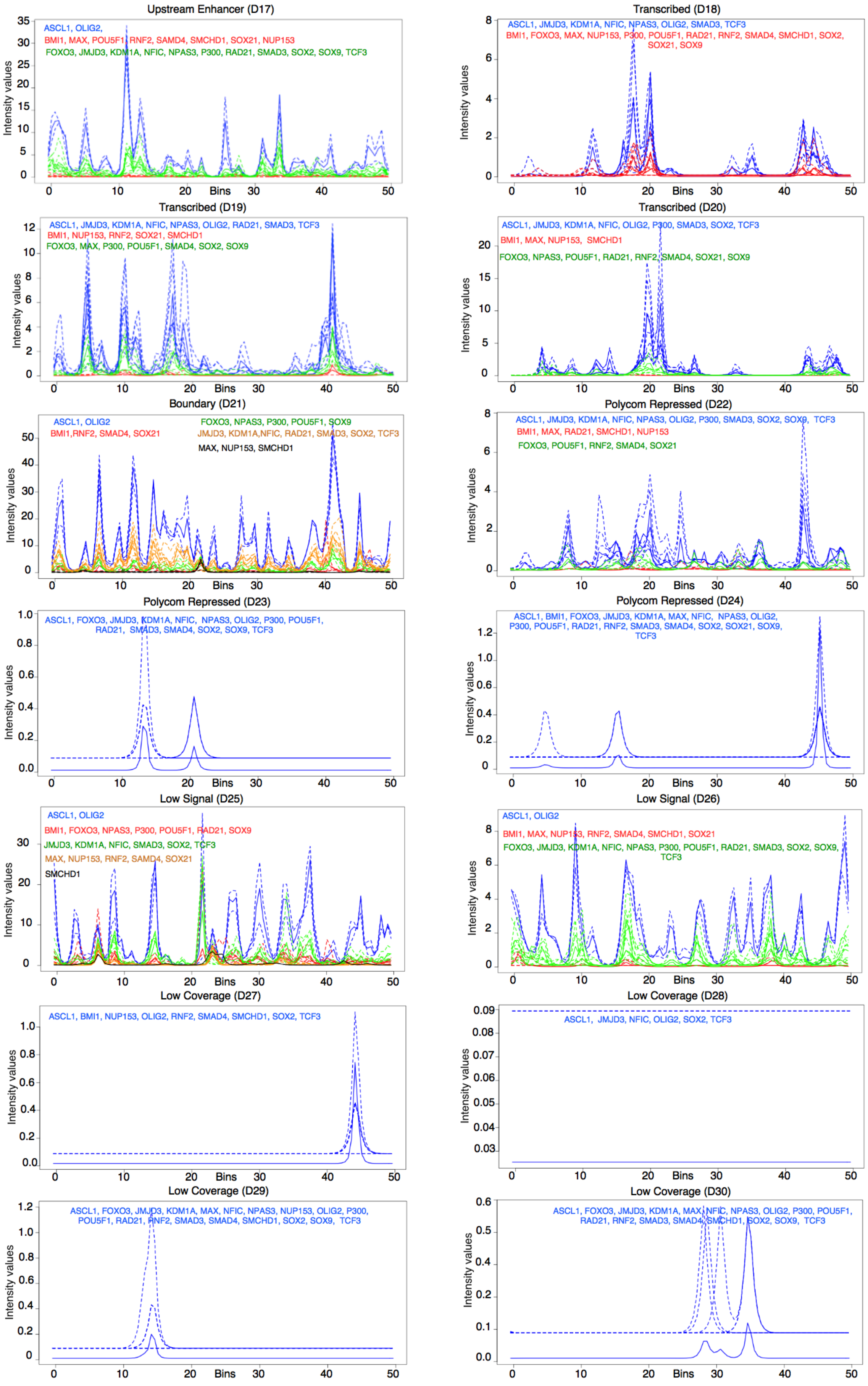

Supplementary Figure S7: Estimated cluster intensities in different chromatin states.

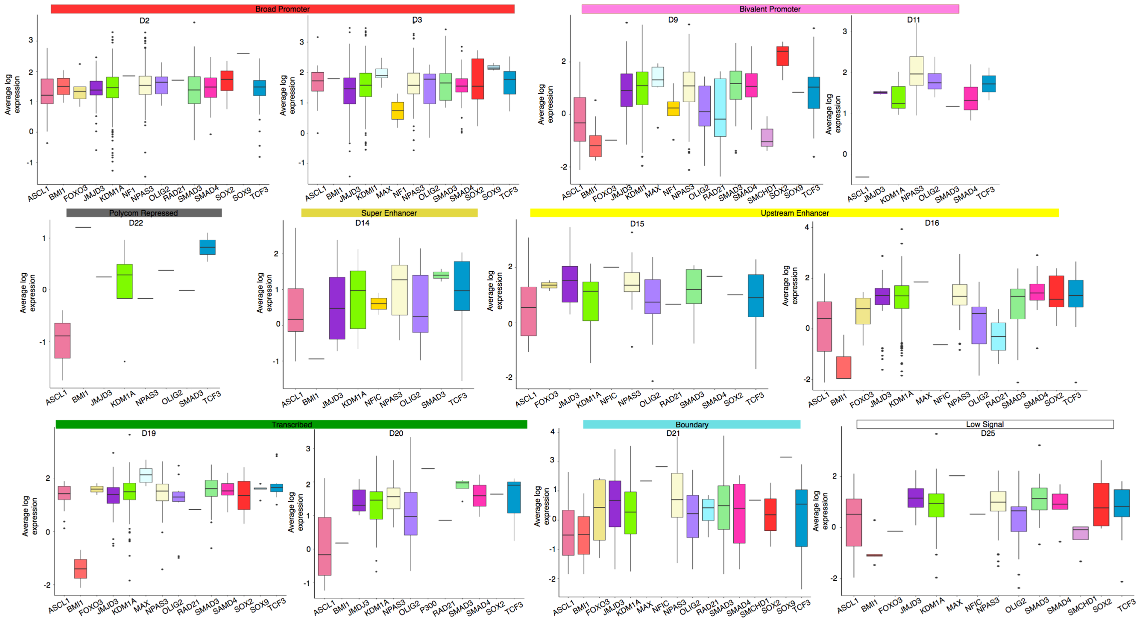

Supplementary Figure S8: Gene expression comparison for individual proteins among different chromatin states.

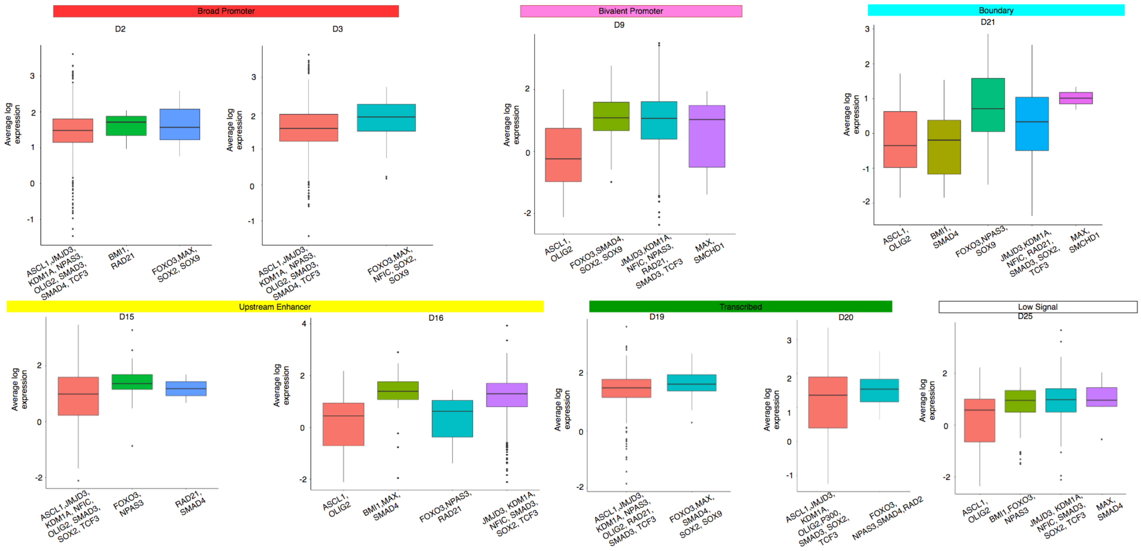

Supplementary Figure S9: Gene expression comparison for predicted clusters among different chromatin states.

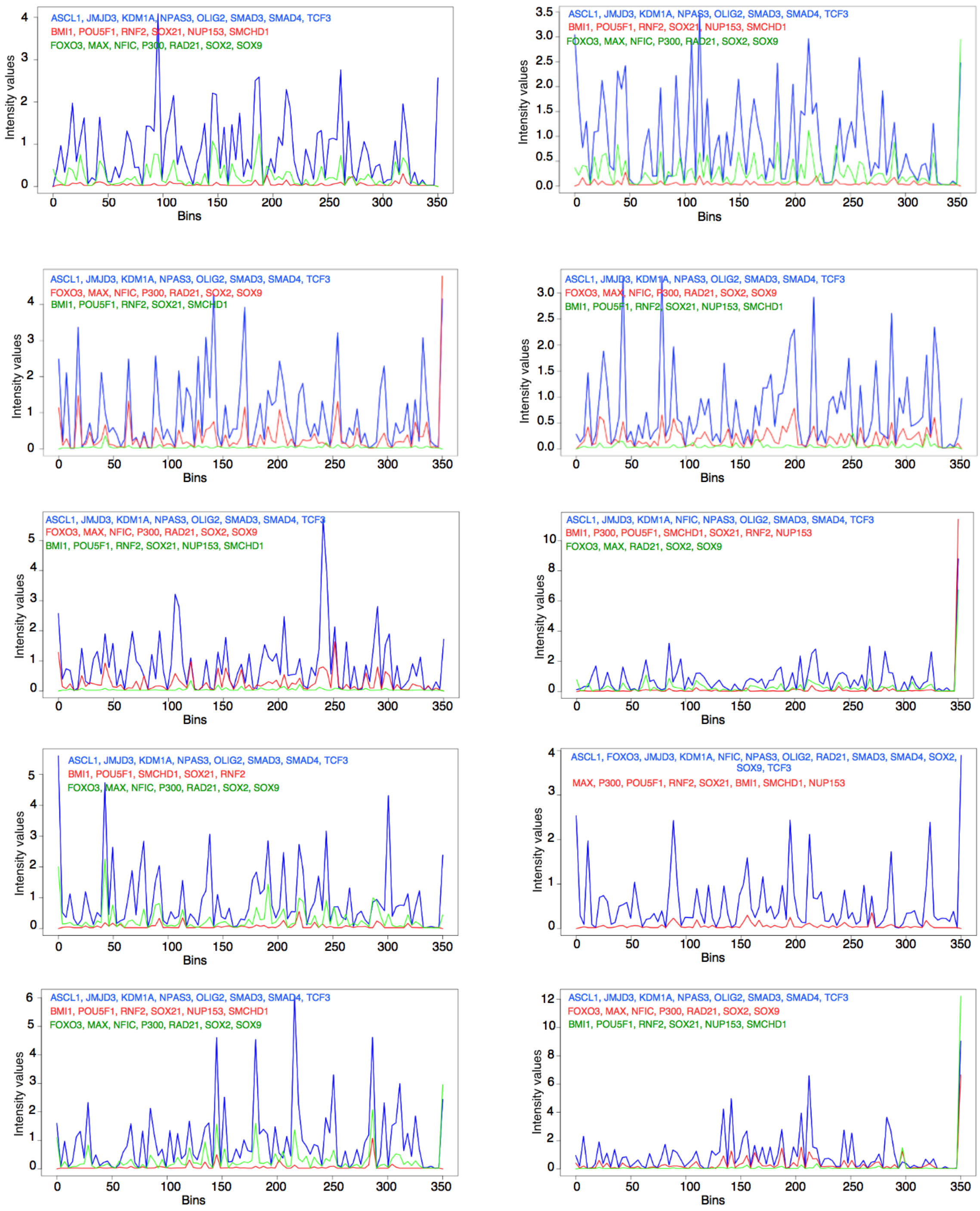

Supplementary Figure S10: Estimated intensity curves of the identified clusters in 10 sub-samples, each with 1,000 randomly selected windows in a Broad Promoter domain (D5).

We have shown the error rates (calculated in the same way as in Subsection 5.1) in Supplementary Table S4.

5.3 Effect of permutation of peaks

We selected a specific region on Chromosome X spanning from 109933423 to 143803727 (Figure S2C) as an example and showed the result in Figure S2D, where we had observed two distinct binding patterns for the cluster consisting of JMJD3, SMAD3 and the cluster consisting of OLIG2. Next, we shuffled all the binding locations within this region, randomly assigned a set of peaks to each protein and performed clustering on this new randomized set of locations. Repeating this procedure 1000 times, we calculated the p-value of observing two distinct clusters with JMJD3-SMAD3 in one cluster and OLIG2 in another cluster under the null hypothesis of all proteins sharing the same binding intensity. We examined the null distribution of the clustering pattern from all 1000 shuffled data and compared it with the clustering result generated from the observed locations. We found that the clusters obtained with the real data are not resulted by chance, but reflect the true structure underlying the protein-DNA bindings.

6 Comparison of clustering results to other methods

7 Time complexity analysis

1. **Chromatin state identification performance**: diHMM was run to identify chromatin states with default parameter setting of 30 nucleosome and domain levels, using neural stem cells data with eight histone marks and CTCF binarized BAM files. This analysis was conducted on a 24-core Haswell-EP E5-2680 v3 workstation with 2.50GHz dual processor node and 128 GB RAM. It took about ten days to complete the analysis.

2. **Clustering performance**: DPM-LGCP was applied to detect the clustering pattern of proteins binding on different chromatin states identified by diHMM. For a total of 21 proteins and 30 chromatin states, it took about two and a half hours to complete the analysis.

References

[1] M. Cha and Q. Zhou, “Detecting clustering and ordering binding patterns among transcription factors via point process models,” *Bioinformatics*, vol. 30, no. 16, pp. 2263–2271, 2014.

[2] H. Rue, S. Martino, and N. Chopin, “Approximate Bayesian inference for latent Gaussian models by using integrated nested Laplace approximations,” *Journal of the Royal Statistical Society: Series B (Statistical Methodology)*, vol. 71, no. 2, pp. 319–392, 2009.

[3] F. Lindgren, H. Rue, and J. Lindström, “An explicit link between Gaussian fields and Gaussian Markov random fields: the stochastic partial differential equation approach,” *Journal of the Royal Statistical Society: Series B (Statistical Methodology)*, vol. 73, no. 4, pp. 423–498, 2011.

[4] D. Simpson, J. B. Illian, F. Lindgren, S. H. Sørbye, and H. Rue, “Going off grid: Computationally efficient inference for log-Gaussian Cox processes,” *Biometrika*, vol. 103, no. 1, pp. 49–70, 2016.

[5] B. M. Taylor and P. J. Diggle, “INLA or MCMC? A tutorial and comparative evaluation for spatial prediction in log-Gaussian Cox processes,” *Journal of Statistical Computation and Simulation*, vol. 84, no. 10, pp. 2266–2284, 2014.

[6] S. A. Lambert, A. Jolma, L. F. Campitelli, P. K. Das, Y. Yin, M. Albu, X. Chen, J. Taipale, T. R. Hughes, and M. T. Weirauch, “The human transcription factors,” *Cell*, vol. 172, no. 4, pp. 650–665, 2018.

[7] J. L. Mateo, D. L. van den Berg, M. Haeussler, D. Drechsel, Z. B. Gaber, D. S. Castro, P. Robson, Q. R. Lu, G. E. Crawford, P. Flicek, *et al.*, “Characterization of the neural stem cell gene regulatory network identifies olig2 as a multifunctional regulator of self-renewal,” *Genome Research*, vol. 25, no. 1, pp. 41–56, 2015.

[8] B. Langmead and S. L. Salzberg, "Fast gapped-read alignment with bowtie 2," *Nature Methods*, vol. 9, no. 4, pp. 357–359, 2012.

[9] H. Li, B. Handsaker, A. Wysoker, T. Fennell, J. Ruan, N. Homer, G. Marth, G. Abecasis, and R. Durbin, "The sequence alignment/map format and samtools," *Bioinformatics*, vol. 25, no. 16, pp. 2078–2079, 2009.

[10] J. Feng, T. Liu, B. Qin, Y. Zhang, and X. S. Liu, "Identifying chip-seq enrichment using macs," *Nature Protocols*, vol. 7, no. 9, pp. 1728–1740, 2012.

[11] C. L. Araya, T. Kawli, A. Kundaje, L. Jiang, B. Wu, D. Vafeados, R. Terrell, P. Weissdepp, L. Gevirtzman, D. Mace, *et al.*, "Regulatory analysis of the c. elegans genome with spatiotemporal resolution," *Nature*, vol. 512, no. 7515, pp. 400–405, 2014.

[12] B. Alberts, D. Bray, K. Hopkin, A. Johnson, J. Lewis, M. Raff, K. Roberts, and P. Walter, *Essential Cell Biology.* Garland Science, 2013.

[13] B. Li and C. N. Dewey, "Rsem: accurate transcript quantification from rna-seq data with or without a reference genome," *BMC Bioinformatics*, vol. 12, no. 1, p. 323, 2011.

[14] A. S. Bais, N. Kaminski, and P. V. Benos, "Finding subtypes of transcription factor motif pairs with distinct regulatory roles," *Nucleic Acids Research*, vol. 39, no. 11, pp. e76–e76, 2011.

[15] T. L. Bailey, N. Williams, C. Misleh, and W. W. Li, "Meme: discovering and analyzing dna and protein sequence motifs," *Nucleic Acids Research*, vol. 34, no. suppl_2, pp. W369–W373, 2006.

[16] S. Heinz, C. Benner, N. Spann, E. Bertolino, Y. C. Lin, P. Laslo, J. X. Cheng, C. Murre, H. Singh, and C. K. Glass, "Simple combinations of lineage-determining transcription factors prime cis-regulatory elements required for macrophage and b cell identities," *Molecular cell*, vol. 38, no. 4, pp. 576–589, 2010.

[17] M.-R. Pan, G. Peng, W.-C. Hung, and S.-Y. Lin, "Monoubiquitination of h2ax protein regulates dna damage response signaling," *Journal of Biological Chemistry*, vol. 286, no. 32, pp. 28599–28607, 2011.

[18] C. Estarás, N. Akizu, A. García, S. Beltrán, X. de la Cruz, and M. A. Martínez-Balbás, "Genome-wide analysis reveals that smad3 and jmjd3 hdm co-activate the neural developmental program," *Development*, vol. 139, no. 15, pp. 2681–2691, 2012.

[19] I. F. Tsigelny, V. L. Kouznetsova, N. Lian, and S. Kesari, "Molecular mechanisms of olig2 transcription factor in brain cancer," *Oncotarget*, vol. 7, no. 33, p. 53074, 2016.

[20] X. Fang, J.-G. Yoon, L. Li, W. Yu, J. Shao, D. Hua, S. Zheng, L. Hood, D. R. Goodlett, G. Foltz, *et al.*, "The sox2 response program in glioblastoma multiforme: an integrated chip-seq, expression microarray, and microrna analysis," *BMC genomics*, vol. 12, no. 1, p. 11, 2011.

[21] L. Xu, C. Alarcón, S. Çöl, and J. Massaguè, "Distinct domain utilization by smad3 and smad4 for nucleoporin interaction and nuclear import," *Journal of Biological Chemistry*, vol. 278, no. 43, pp. 42569–42577, 2003.

[22] M. Goolam, A. Scialdone, S. J. Graham, I. C. Macaulay, A. Jedrusik, A. Hupalowska, T. Voet, J. C. Marioni, and M. Zernicka-Goetz, "Heterogeneity in oct4 and sox2 targets biases cell fate in 4-cell mouse embryos," *Cell*, vol. 165, no. 1, pp. 61–74, 2016.

[23] I. Imayoshi and R. Kageyama, "bhlh factors in self-renewal, multipotency, and fate choice of neural progenitor cells," *Neuron*, vol. 82, no. 1, pp. 9–23, 2014.

[24] H.-B. Yu, R. Johnson, G. Kunarso, and L. W. Stanton, "Coassembly of rest and its cofactors at sites of gene repression in embryonic stem cells," *Genome research*, vol. 21, no. 8, pp. 1284–1293, 2011.

[25] S. Tanaka, Y. Kamachi, A. Tanouchi, H. Hamada, N. Jing, and H. Kondoh, "Interplay of sox and pou factors in regulation of the nestin gene in neural primordial cells," *Molecular and cellular biology*, vol. 24, no. 20, pp. 8834–8846, 2004.

[26] A. Nitzsche, M. Paszkowski-Rogacz, F. Matarese, E. M. Janssen-Megens, N. C. Hubner, H. Schulz, I. de Vries, L. Ding, N. Huebner, M. Mann, *et al.*, "Rad21 cooperates with pluripotency transcription factors in the maintenance of embryonic stem cell identity," *PloS one*, vol. 6, no. 5, p. e19470, 2011.

[27] C. Bradney, M. Hjelmeland, Y. Komatsu, M. Yoshida, T.-P. Yao, and Y. Zhuang, "Regulation of e2a activities by histone acetyltransferases in b lymphocyte development," *Journal of Biological Chemistry*, vol. 278, no. 4, pp. 2370–2376, 2003.

[28] P.-R. Sudhir and C.-H. Chen, "Proteomics-based analysis of protein complexes in pluripotent stem cells and cancer biology," *International journal of molecular sciences*, vol. 17, no. 3, p. 432, 2016.

Predicted interactions in chromatin states	Interactions mentioned in other works
BMI1 and RNF2 observed in Broad Promoter, Upstream Enhancer, Transcribed, Boundary states	Through proteomic analysis the authors showed that monoubiquitination of H2AX mediated by the RNF2-BMI1 complex is critical for the efficient formation of y-2AX and functions as a proximal regulator in DNA damage response [17]
JMJD3, SMAD3 observed in Broad Promoter, Bivalent Promoter, Super Enhancer, Upstream Enhancer, Transcribed states	Through ChIP-Seq and expression analysis the authors showed that JMJD3 and SMAD3 co-localize at the TSS of TGF-β responsive genes in NSCs [18]
OLIG2, KDM1A, TCF3 observed in Broad Promoter, Super Enhancer, Upstream Enhancer, Transcribed, Polycomb Repressed states	Using a computational tool VisANT, the authors showed that KDM1A is involved in de-methylation of p300 HAT which activates OLIG2 through TCF3 [19]
NPAS3, SOX2 observed in Upstream Enhancer, Poised Enhancer states	Through mass spectrometry in neural stem cells the authors showed the interactions among important TFs in NSC, among which are SOX2 and NPAS3 [20]
NUP153, SMAD4 observed in Upstream Enhancer state	The authors studied the nuclear import mechanisms of SMAD3 and SMAD4 and found that the C-terminal MH2 domain of SMAD3 had the primary nuclear import activity allowing direct contact with FG-repeat containing CAN/Nup214 and NUP153 [21]
POU5F1, SOX21 observed in Broad Promoter, Super Enhancer, Upstream Enhancer,Transcribed, Polycomb Repressed states	Using single-cell transcriptomics the authors showed that SOX21 expression might be regulated by heterogeneous POU5F1 and/or SOX2 activity in the embryo [22]
P300, SMAD family observed in Bivalent Promoter, Super Enhancer states	The authors showed that during astrocyte fate determination Activated SMAD family works with STAT3, which is then mediated by P300/CBP resulting in the induction of astrocytic gene expression [23]
NFIC, SOX, FOX and Basic helix-loop-helix (bHLH) family observed in Broad Promoter, Bivalent Promoter, Super Enhancer states	Through DNase-seq in combination with analysis of histone modifications, ChIP-seq and gene expression analysis in mouse neural stem cells, the authors identified these TFs as crucial members of cis-regulatory network [7]
POU5F1, SOX2 observed in Bivalent Promoter, Transcribed states	Through chromatin immunoprecipitation coupled with DNA microarrays and genome-wide mapping of OCT4 and SOX2 sites in human ES cells, the authors showed that they co-regulate multiple genes [24, 25]
RAD21, POU5F1, SOX2 observed in Broad Promoter, Super Enhancer, Upstream Enhancer, Transcribed states	Through ChIP-seq analyses the authors show that RAD21 has specific cohesin binding pattern that is characterized by CTCF independent co-localization of cohesin with pluripotency related transcription factors POU5f1 and SOX2 among others [26]
TCF3, P300 observed in Super Enhancer, Upstream Enhancer states	Through gel filtration and co-immunoprecipitation analysis of human pre-B cell nuclear extract, the authors showed that E2A co-eluted with the P300, CBP, and PCAF [27]
POU5F1, RNF2 observed in Broad Promoter, Super Enhancer, Upstream Enhancer states	Through CoIP/IP, the authors showed the interactions between POU5F1 and RNF2 in embryonic stem cells [28]

Supplementary Table S1: Known interactions predicted by the algorithm

Initial number of clusters	Estimated error rate
2	0.2
3-21	0.0

Supplementary Table S4: Error rates of clustering on a Broad Promoter domain with different number of initial clusters.

Chromatin state	DPM-LGCP	K-means	CLARANS
Bivalent Promoter (D8)	(1) ASCL1, JMJD3, KDM1A, NFIC, NPAS3, OLIG2, SMAD3, TCF3; (2) BMI1, FOXO3, MAX, P300, POUF51, RAD21, RNF2, SMAD4, SMCHD1, SOX2, SOX21, SOX9	(1) ASCL1, KDM1A, OLIG2, TCF3; (2) BMI1, FOXO3, JMDJ3, MAX, NFIC, NPAS3, P300, POU5F1, RAD21, RNF2, SMAD3, SMAD4, SMCHD1, SOX2, SOX21, SOX9	(1) ASCL1, KDM1A, OLIG2, TCF3; (2) BMI1, FOXO3, JMJD3, MAX, NFIC, NPAS3, P300, POU5F1, RAD21, RNF2, SMAD3, SMAD4, SMCHD1, SOX2, SOX21, SOX9
Super Enhancer (D14)	(1) ASCL1, JMJD3, KDM1A, NFIC, NPAS3, OLIG2, P300, SMAD3, SOX2, SOX9, TCF3; (20 BMI1, MAX, NUP153, SMCHD1; (3) FOXO3, POU5F1, RAD21, RNF2, SMAD4, SOX21	ASCL1, JMDJ3, KDM1A, NFIC, NPAS3, OLIG2, P300, SMAD3, SOX2, SOX9, TCF3; (2) BMI1, FOXO3, MAX, POU5F1, RAD21, RNF2, SMAD4, SOX21, NUP153, SMCHD1	(1) ASCL1, FOXO3, JMJD3, KDM1A, NFIC, NPAS3, OLIG2, P300, POU5F1, RNF2, SMAD3, SOX2, SOX9, TCF3; (2) BMI1, MAX, RAD21, SMAD4, SOX21, NUP153, SMCHD1
Upstream Enhancer (D15)	(1) ASCL1, JMJD3, KDM1A, NFIC, OLIG2, SMAD3, SOX2, TCF3; (2) BMI1, NUP153, SMCHD1; (3) FOXO3, NPAS3, P300, POU5F1, RNF2, SOX9; (4) MAX, RAD21, SMAD4, SOX21	ASCL1, FOXO3, JMDJ3, KDM1A, NFIC, NPAS3, OLIG2, P300, POU5F1, SMAD3, SOX2, SOX9, TCF3; (2) BMI1, MAX, NUP153, RAD21, RNF2, SMAD4, SMCHD1, SOX21	(1) ASCL1, FOXO3, JMJD3, KDM1A, NFIC, NPAS3, OLIG2, P300, POU5F1, RNF2, SMAD3, SOX2, SOX9, TCF3; (2) BMI1, MAX, NUP153, RAD21, SMAD4, SMCHD1, SOX21
Polycomb Repressed (D22)	(1) ASCL1, JMJD3, KDM1A, NFIC, NPAS3, OLIG2, P300, SMAD3, SOX2, SOX9, TCF3; (2) BMI1, MAX, RAD21, SMCHD1, NUP153; (3) FOXO3, POU5F1, RNF2, SMAD4, SOX21;	ASCL1, JMDJ3, KDM1A, NFIC, OLIG2, P300, SMAD3, SOX2, SOX9, TCF3; (2) BMI1, FOXO3, MAX, NPAS3, POU5F1, RAD21, RNF2, SMAD4, SOX21, SMCHD1, NUP153	(1) ASCL1, JMJD3, KDM1A, NFIC, OLIG2, P300, SMAD3, SOX2, SOX9, TCF3; (2) BMI1, FOXO3, MAX, NPAS3, POU5F1, RAD21, RNF2, SMAD4, SOX21, SMCHD1, NUP153
Transcribed (D18)	(1) ASCL1, JMJD3, KDM1A, NFIC, NPAS3, OLIG2, SMAD3, TCF3; (2) BMI1, FOXO3, P300, POU5F1, RNF2, SMAD4, SOX2, SOX2, SOX9 MAX, RAD21, SMCHD1, NUP153;	(1) ASCL1, JMDJ3, KDM1A, NFIC, NPAS3, OLIG2, SMAD3, TCF3; (2) BMI1, FOXO3, MAX, P300, POU5F1, RAD21, RNF2, SMAD4, SOX21, SMCHD1, NUP153 SOX2, SOX9	(1) ASCL1, JMJD3, KDM1A, NFIC, NPAS3, OLIG2, SMAD3, TCF3, ; (2) FOXO3, MAX, P300, POU5F1, RNF2, SMAD4, SOX2, SOX21, SOX9, RAD21, SMCHD1, BMI1, NUP153
Boundary (D21)	(1) ASCL1, OLIG2; (2) FOXO3, NPAS3, P300, POU5F1, SOX9; (3) BMI1, RNF2, SMAD4, SOX21; (4) JMJD3, KDM1A, NFIC, RAD21, SMAD3, SOX2, TCF3; (5) MAX, NUP153, SMCHD1	ASCL1, FOXO3, JMDJ3, KDM1A, NFIC, NPAS3, OLIG2, P300, SMAD3, SOX2, TCF3; (2) BMI1, MAX, POU5F1, RAD21, RNF2, SMAD4, SOX21, SMCHD1, NUP153, SOX9	(1) ASCL1, JMJD3, KDM1A, NFIC, NPAS3, OLIG2, P300, RAD21, SMAD3, SOX2; (2) BMI1, FOXO3, MAX, NUP153, POU5F1, RNF2, SMAD4, SMCHD1, SOX21, SOX9

Supplementary Table S5: Comparison of clustering results with K-means and CLARANS
